# Predicting emotional valence in autism: a preregistered study in the Bayesian Brain framework

**DOI:** 10.1186/s13229-026-00730-3

**Published:** 2026-07-21

**Authors:** Irene Sophia Plank, Alexandra Pior, Anna Yurova, Julia Nowak, Zhuanghua Shi, Christine M. Falter-Wagner

**Affiliations:** 1 Department of Psychiatry and Psychotherapy, LMU University Hospital, LMU Medizin, Ludwig-Maximilians-Universität München, Nußbaumstraße 7, Munich, 80336 Germany; 2 Department of Sociology, Faculty of Behavioral and Social Sciences, University of Groningen, Grote Rozenstraat 1/2, Groningen, 9712 TS Netherlands; 3 Department of Psychology, Faculty of Psychology and Neuroscience, Maastricht University, Universiteitssingel 40, Maastricht, 6229 ER Netherlands; 4 Department of Psychology, LMU Munich, Leopoldstr. 13, 80802 Munich, Germany; 5 NeuroImaging Core Unit Munich (NICUM), LMU Munich, Nußbaumstraße 7, Munich, 80336 Germany

**Keywords:** Probabilistic associative learning, Hierarchical gaussian filter, Autism spectrum disorder, Volatility, Predictive coding, Bayesian brain framework

## Abstract

**Background:**

Autism spectrum disorder (ASD) affects social interaction, communication and behavioural flexibility. Recent theories grounded in the Bayesian Brain Framework propose that autistic individuals process environmental uncertainty differently, overweighting volatility when updating predictions about the world. However, the robustness of these predictive processing differences and their relevance to core symptoms remains unclear.

**Methods:**

Lawson and colleagues [1] reported a tendency to overestimate environmental volatility in autistic adults based on belief states extracted using a Hierarchical Gaussian Filter. We extended their paradigm to a domain central to the autistic experience: rather than distinguishing houses from faces, participants detected valence in emotional facial expressions. Preregistered hypotheses were evaluated using Bayesian linear mixed models based on data of 22 autistic and 22 non-autistic participants.

**Results:**

Despite using an identical computational model to extract belief states, we did not find robust differences between autistic and non-autistic adults, though autistic participants showed a non-credible trend towards increased processing of phasic volatility. Largely comparable probabilistic associative learning was also reflected in response times and pupil sizes.

**Limitations:**

While participants reacted faster to expected than unexpected trials in the beginning, this effect decreased over the course of the experiment; thus, possibly indicating that the individual blocks of stable cue-outcome associations were too short.

**Conclusions:**

We were unable to find credible differences in environmental volatility and learning rate updates using a task designed to probe a key autistic difficulty, facial emotion recognition. The current results call into question the generalisability and clinical relevance of prior findings.

**Supplementary Information:**

The online version contains supplementary material available at 10.1186/s13229-026-00730-3.

## Introduction

In a dynamic world with continuous changes, the human brain constantly tries to predict what is going to happen next to optimise neural resource deployment. The Bayesian Brain framework conceptualises predictive processing based on the integration of a probability distribution, capturing the expectations as the prior, which is combined with current sensory input to update the expectation, i.e. the posterior [2]. Applications of the framework to Autism Spectrum Disorder (ASD) have been very promising [3–7]. ASD is a neurodevelopmental disorder characterised by two symptom clusters: difficulties in social interactions and communication and restrictive or repetitive interests and behaviour [8]. Central to the Bayesian Brain framework is how the system responds when predictions and incoming sensory input diverge. This mismatch results in a prediction error weighted by the estimated precision of the input, determining the extent to which predictions are updated [2]. However, the update depends not only on the precision of the input but also on the perceived stability of the environment. This environmental volatility can be separated into tonic and phasic components to distinguish between transient noise and sudden environmental changes [9]. Tonic volatility represents the stable, background level of change that is expected to persist over time, acting as a baseline expectation of environmental stability, where increased levels of tonic environmental volatility reflect a belief that the environment is inherently prone to frequent change [1]. In contrast, phasic volatility refers to the dynamic and situational fluctuations in uncertainty that occur in response to specific, surprising events [9]. Optimal learning requires a balance between tonic and phasic volatility to appropriately adjust behaviour to the underlying stability of the world. In the early 2010s, Pellicano and Burr [5] proposed that both hyper- and hyposensitivity in the same autistic individual can be explained by hypo-priors, i.e., less precise priors, resulting in autistic people relying more heavily on sensory input due to decreased influence of the prior. In response, Brock [3] noted that this pattern could be explained by both top-down influences, i.e., hypo-priors, or bottom-up influences like reduced sensory noise resulting in an increased weight attributed to sensory input. Subsequently, theories were proposed considering hierarchical levels to reflect the cortical hierarchy central to predictive coding, the neurobiological formulation of the Bayesian Brain framework. Van de Cruys and colleagues [7] proposed that autistic individuals inflexibly attribute higher weight to prediction errors. Van de Cruys and colleagues argued that this pattern explains a wide array of symptoms, including deficits in executive functioning and social cognition. Lawson and colleagues [4] emphasised that precision develops dynamically on different hierarchical levels and proposed that symptoms of autism can be explained by an imbalance of precision attributed to the sensory input relative to the priors [4], specifically on higher levels of the hierarchy [10]. In a recent meta-analysis, these accounts were summarised under the label “imbalance hypothesis” with the authors arguing all of them proposing imbalances leading to a greater reliance on sensory inputs [11]. This meta-analysis found mixed results regarding the integration of priors possibly to low power and inconsistent approaches, but various studies showed differences in learning between autistic and non-autistic individuals [11]. Recently, Shi et al. [5] proposed the atypical iterative prior updating account following which autistic individuals integrate prior knowledge similarly to non-autistic individuals for immediate perceptual judgements yet revise their priors more dynamically on a trial-by-trial basis, placing greater weight on new sensory information when updating beliefs about the environment. In their central tendency task, this elevated update rate initially weakens reliance on accumulated priors, yielding reduced central tendency early in learning, but converges with the performance of non-autistic individuals as stable expectations accrue. This pattern suggests that priors form typically in ASD, despite overweighting of sensory information in believe updating. Most research focusing on predictive processing in ASD used tasks not directly capturing core symptoms of autism. For instance, Lawson et al. [9] adapted a probabilistic associative learning task to experimentally manipulate changes in learned expectations and sensory noise to assess effects thereof on behaviour in a non-social context. In their binary classification task, participants had to differentiate between a house or a human face (see also [12]). The visual stimuli were preceded by either a low or a high tone, which was predictive of the next decision. In the task designed by Lawson and colleagues [1], learning occurred on three levels simultaneously: the level of the stimulus, the level of the cue-outcome association and the level of the environment. They used computational modelling based on participants’ reaction times to extract participant-specific estimates of perception, specifically tonic volatility and learning rates, and estimates of how the perception influences the response in the form of phasic volatility and outcome surprise. While both groups learned the task equally well overall, adults with ASD seemed to overestimate environmental volatility and produced responses more strongly governed by outcome surprise [1]. They complemented their investigation of estimates based on reaction times with pupillometry to indirectly capture phasic noradrenaline function. The pupil sizes revealed increased encoding of surprise in pupillary responses. Results such as these emphasize the ways in which the Bayesian brain framework can be used in increasing the understanding of ASD by allowing us to disentangle different mechanisms in more detailed ways. Specifically, if the reported overestimation of environmental volatility applies to socio-emotional contexts, this mechanism could explain difficulties with emotion recognition associated with ASD [13]. There have been several other studies investigating volatility in autism. Sapey-Triomphe and colleagues [14, 15] also used a probabilistic associative learning task but found no differences in the levels of tonic volatility parameters extracted with the HGF between autistic and non-autistic adults, at odds with Lawson et al. [1]. Regardless of comparable tonic volatility parameters, they found neural activation associated with high level predictions governed by the participant’s belief regarding environmental tonic volatility was increased in autistic adults compared to non-autistic adults in the orbitofrontal cortex, the posterior cingulate cortex and the posterior superior temporal sulcus [15]. Thus, while neural correlates of predictive processing might differ between autistic and non-autistic adults, these differences are not necessarily reflected in the volatility parameters extracted from their behaviour. Furthermore, no differences in tonic volatility extracted from the behaviour were found in the Wisconsin card-sorting task [16] or grip force in a size weight illusion task [17]. However, Arthur and colleagues [17] found decreased influence of tonic volatility based on gaze pitch angle in autistic compared to non-autistic adults when playing tennis in virtual reality.

These results show a mixed picture of tonic volatility modifications in autism, possibly due to the differences in models and tasks [11]. This mixed picture stresses the necessity of assessing the robustness of reported differences in predictive processes between autistic and non-autistic adults. Thus, the main aim of the current study was to assess whether reported differences in stimulus uncertainty and environmental volatility between autistic and non-autistic adults in the seminal study by Lawson and colleagues [1] extend to relevant symptom areas, specifically socio-emotional processing. Despite the elegant design developed by Lawson et al. [1], the stimuli and task used, differentiating between house and face stimuli, bear little relevance for either autistic core symptoms or navigating real life in general. Hence, while we did stick to the design from Lawson and colleagues [1, 12] allowing to manipulate not only stimulus and associative uncertainty but also volatility separately, we adapted the task to induce associations between tone cues and emotional facial expressions while participants were asked to judge the valence of these expressions. While this simple decision limits generalisability to the complexity of emotion recognition in everyday life, this adaptation allowed us to keep the probabilistic associative learning process implemented in the task, i.e., we used the same trial structure as well as the same probabilities and volatility manipulation. This adaptation of decision but not task structure allowed us to directly compare the current results to the results of Lawson and colleagues [1].

To characterise each participant’s probabilistic learning process, we modelled reaction times using the same three-level Hierarchical Gaussian Filter HGF, [9, 18] as Lawson and colleagues [1]. We followed a Bayesian workflow for both computational models and conventional analyses [19, 20]. Such a workflow is necessary for robust modelling results but still not routinely followed, possibly contributing to the mixed results regarding modified Bayesian Brain processes in ASD [11]. We preregistered hypotheses mirroring the findings of Lawson and colleagues [1] in a socio-emotional context: an increased estimated phasic volatility and environmental tonic volatility as well as an increased learning rate update for environmental changes and a decreased learning rate update for cue-outcome probabilities in autistic compared to non-autistic adults. Furthermore, we assessed reaction times and pupil sizes with conventional analyses similar to Lawson and colleagues [1], expecting previously reported differences in the effect of expectancy on both measurements between autistic and non-autistic adults.

## Method

The current study was preregistered prior to data collection on the Open Science Framework: https://osf.io/f23nq. The preregistration describes three groups (ADHD, ASD and comparison participants). Because model comparison indicated different best-fitting models, data of ADHD and an explorative ASD+ADHD group were analysed and reported separately to ensure clarity and interpretability [21]. All models which were not preregistered are labelled as explorative. Data preprocessing and analysis was performed in MATLAB *R2023a* [22], Python *3.9.2* in Spyder *6.0.3* [23] and R *4.3.3* [24] in RStudio [25]. The paradigm was programmed in Psychtoolbox-3 [26, 27]. All code as well as anonymised data can be found here: https://github.com/IreneSophia/EMBA_PAL.

### Sample

The current study is part of a larger project investigating facial emotion recognition in adults with ASD and Attention-Deficit/Hyperactivity Disorder (ADHD), with other results reported elsewhere. The project was approved by the Ethics committee of the Medical Faculty, LMU Munich (reference number: 22–0561) and conducted according to the principles expressed in the Declaration of Helsinki. Participants received monetary compensation for their time and transportation costs, if applicable. Inclusion criteria across groups were sufficient knowledge of German to understand the task instructions, estimated intelligence quotient (IQ) above 70, normal or corrected-to-normal vision, magnetic resonance imaging possible, no neurological diagnoses, no reported acute suicidality, valid consent and being between 16 and 45 years old. Additionally, participants in the comparison group had no psychiatric diagnosis and took no psychotropic medication, while autistic participants had a formal diagnosis of ASD but not ADHD. We asked autistic participants whether they prefer identity-first or person-first language (identity-first: 37%; person-first: 37%; no preference: 19%; no answer: 7%); since there was no clear consensus, we will use both identity-first and person-first language throughout the manuscript.

We intended to analyse at least 22 participants per group based on power analyses performed with PANGEA v0.2 [28] to achieve at least 80% power to detect a medium effect of *d* = 0.45 in each of the tasks of the project. We decided to use the same target effect size across tasks in this project and, thus, chose a medium effect size and did not base the effect size on previous reported effects. For the probabilistic learning task used in the current study, we preregistered a power analysis based on a 2 × 3 × 3 mixed design with two within-subject conditions (one with two and one with three levels), 22 participants per group and a total of 336 trials nested in the conditions. This power analysis resulted in 94% power to detect a difference between two groups, exceeding our goal of 80% power.

We recruited a total of 60 participants for the current study, of which nine were to pilot the task. Of the remaining 51 participants, data of seven participants was excluded following the preregistered plan due to accuracy below 66.7% (two of each group), acute suicidality (one autistic) and change in diagnosis (one autistic participant received an ADHD diagnosis, another was re-evaluated as non-autistic with ADHD). Thus, the final analysed sample consisted of 22 autistic and 22 comparison participants of comparable age and gender as well as handedness distribution (see Table 1). All participants reported that their gender matched their assigned gender at birth. Due to technical difficulties, pupil sizes as a phasic index of norepinephrine functioning [9] were only collected from a smaller sample, resulting in the analysis of data from 18 participants per group.

**Table 1:** Sociodemographic and clinical self-assessment in the two groups

measurement	ASD	COMP	log(BF)
Age	29.86 ± 7.62 (20 to 45)	27.59 ± 5.86 (21 to 42)	-0.971
Age at diagnosis	23.42 ± 10.33 (4 to 43)		
Education	3.77 ± 1.02 (2 to 5)	4.36 ± 0.85 (2 to 5)	0.583
Gender	11 women – 11 men	10 women – 12 men	-0.980
Handedness	1 ambidextrous – 3 lefthanded – 17 righthanded	3 lefthanded – 19 righthanded	-2.445
IQ estimate	114.11 ± 15.14 (78 to 144)	109.14 ± 8.62 (92 to 131)	-0.494
RADS-R	148.77 ± 40.44 (55 to 217)	46.50 ± 36.01 (9 to 142)	17.801*

Note. This table shows means and standard deviations as well as range in parentheses. Education was reported on a scale from 1 = “no educational or vocational training” to 5 = “University degree”, while gender was captured with free text allowing for self-identification. Handedness of one autistic participant as well as age at diagnosis of three autistic participants are missing. Asterisks denote credible group differences based on Bayesian ANOVAs or contingency tables for gender distribution. IQ = intelligence quotient; RADS-R = Ritvo autism diagnostic scale-revised

### Procedure

Data collection started with participants giving written informed consent and filling out a demographic questionnaire as well as the Beck Depression Inventory (BDI, [29]) to check for acute suicidality. Next, they performed a verbal and non-verbal IQ assessment (MWT-B, [30], CFT 20-R, [31]) as well as a questionnaire assessing ASD symptom severity (RADS-R, [32]). Then, participants performed four behavioural tasks in counterbalanced order, which took around 60 min in total. The results of all tasks but the probabilistic learning task as well as questionnaires relating to those other tasks are reported elsewhere [21, 33, 34]. All tasks were performed on a Lenovo Legion Pro 5 laptop which was mounted in a laptop stand (resolution: 2560 × 1600 pixel; size: 344 × 215 mm; refresh rate: 60 Hz). For these behavioural tasks, participants were seated in a small, soundproof testing chamber with consistent lighting conditions. The behavioural tasks were followed by 60 min in the MRI scanner to collect structural and functional neuroimaging data reported elsewhere [35]. We used a LiveTrack Lightning eye tracker (Cambridge RS, sampling rate 500 Hz) together with a headrest to ensure a stable viewing distance of 57 cm. Only participants with a successful calibration based on a 9-point calibration (accuracy < 0.5°) were included in the analysis of the eye-tracking data for the current study.

### Stimulus creation

**Fig. 1:**
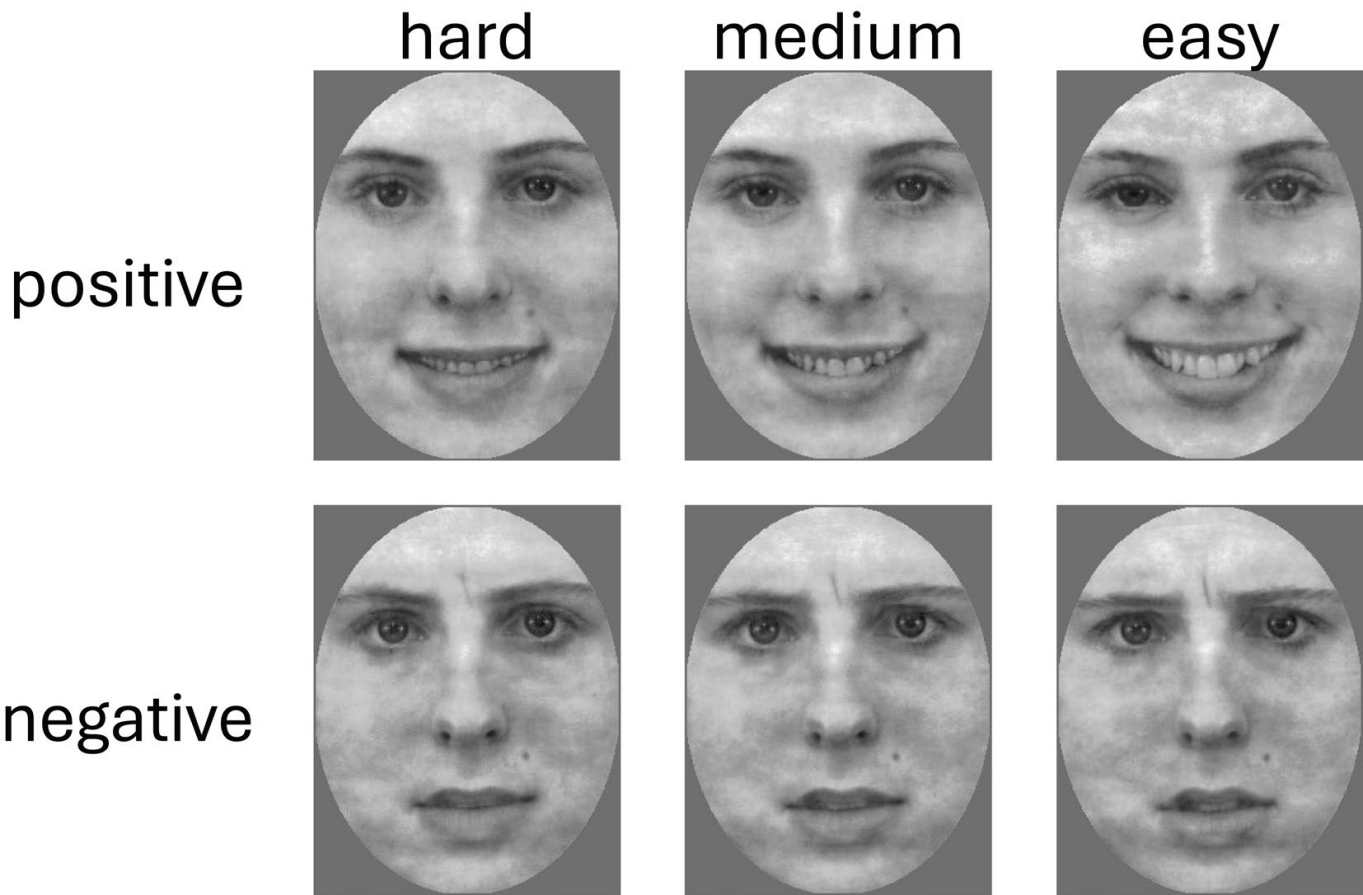
Example stimuli based on the KDEF identity F05. For each identity, six versions were created, each three difficulty levels of the positive (happy) and negative (fearful) emotion

For the stimulus creation, we used pictures of people portraying three expressions from the Karolinska Directed Emotional Faces (KDEF) database [36]: emotionally neutral, fearful and happy. Then, we used WinMorph 3.01 to morph the emotionally neutral face with the fearful and happy facial expressions of the same person, thereby creating videos showing the emotionally neutral face gradually and continually taking on an emotion. These videos were also used in the facial emotion recognition task reported in Plank et al. [33]. The naturalness of these videos was rated on a scale from 0 (not natural) to 100 (very natural) by 21 (male presenting faces) and 25 (female presenting faces) people. Based on these ratings, we selected the following six identities: F02, F05, F09, M10, M11 and M13 (naturalness of selected videos: range: 70.04 to 82.81, mean = 76.55). We took still depictions including differing amounts of the emotional facial expressions, choosing three difficulties for our binary emotional valence recognition task (see Fig. 1): 50% (hard), 75% (medium) and 100% of a specific emotion (easy).

### Probabilistic associative learning task: emotion recognition

**Fig. 2:**
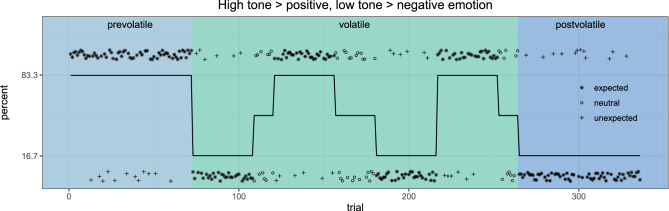
Trial order for the experiment. All participants first encountered a stable, prevolatile phase in which 83.3% of the high tones were followed by an emotionally positive facial expression and thus expected, while 16.7% were unexpectedly followed by an emotionally negative facial expression. In the middle phase, expected associations changed multiple times (volatile phase) and short, neutral blocks had equal associations between the emotions and tones. In the postvolatile phase, high tones were associated with emotionally negative expressions with a stable association for the duration of the phase; thus, this phase was complementary to the prevolatile phase with switched associations

This task was adapted from Lawson et al. [17]. Participants encountered 336 faces in a two-alternative emotional valence recognition task, each of which was presented in the centre of the screen (3.46 degrees wide and 4.72 degrees high). They were asked to decide as fast and accurately as possible whether the valence of the portrayed emotion is positive (happy) or negative (fearful). Answers were logged using two keys in one row of an external numpad which participants were allowed to position in a comfortable way. Whether the left or the right key corresponded to positive was counterbalanced across participants. While associations between spatial side and valence have been reported which differ between handedness [37], the equal distribution of handedness and spatial side corresponding to positive in the current sample make influences on group differences unlikely. Trials had the following structure: a fixation cross was presented in the middle of the screen while a tone was played for 300ms followed by the presentation of the face for 150ms and then a response window during which a fixation cross was presented again but without the tone (jittered duration, uniform distribution, range = 1500 to 1800ms). The tone was predictive of the valence of the emotion portrayed in the following facial expression and served as a cue for the following outcome. The association of cue-outcome changed over the course of the task as shown in Fig. 2. Specifically, the task manipulated the following within-participant factors: *Phase* of the task (stable, prevolatile; volatile; stable, postvolatile), *Expectancy* (expected cue-outcome association, unexpected cue-outcome association), *Difficulty* of the decision (hard, medium, easy), *Valence* (negative: fear, positive: happiness) and *Tone* (high, low). Whether trials were classified as an expected or an unexpected cue-outcome association was determined on the predominant association within blocks of 36 or 72 trials. Additionally, there were two blocks of 12 and one block of 24 trials with neutral probability (0.50) which were dropped from the analysis. Longer blocks with 72 trials of the same cue-outcome probabilities were considered stable compared to the more volatile phase where cue-outcome probabilities changed at least each 36 trials. Over the course of the task, all combinations of the within-participant factors were equally likely. Participants performed eight practice trials before the task, and the total task duration was 12 min. All participants encountered the same trial order.

### Preprocessing of reaction times and pupil sizes

Reaction time analysis was based on correct trials. Furthermore, we excluded extreme reaction times defined by the interquartile method.

We analysed pupil sizes of the left eye as measured as the length of the major axis in mm for all participants where usable data was collected for at least 50% of the task [38]. We preprocessed the data following the recommendations published in Mathôt and Vilotijević [39] and using the DataMatrix package (for details see S2.1). If a participant is missing more than one third of all trials, their data is not included in the analysis.

We did not preregister a specific time window to assess our hypotheses regarding the effect of expectancy on pupil sizes; thus, we use cross-validation to extract samples from the whole response time window as recommended by Mathôt and Vilotijević [39]. Additionally, we explore associations between participant-specific HGF parameters and pupil sizes, comparing averages in the time window 500 to 1500ms for precision-weighted prediction errors on the third level and in the time window 1000 to 1250 for the expectancy effect based on Lawson et al. [9] between ASD and COMP participants.

### Hierarchical gaussian filter

**Fig. 3:**
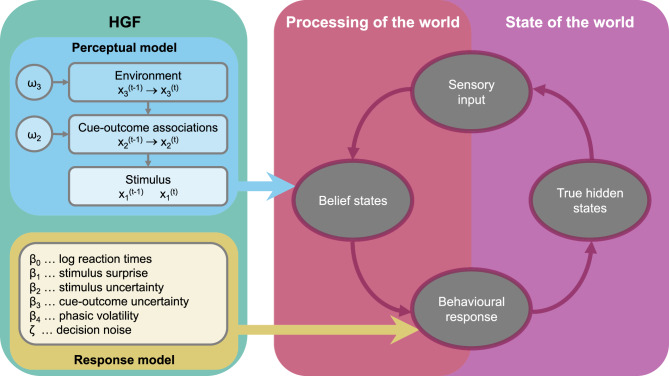
Conceptual visualisation of the perceptual and the response model forming the Hierarchical Gaussian Filter (HGF) and their link to the processing and the state of the world. Predictive processing relies on belief states that estimate the true hidden states of the world based on the sensory input. These belief states govern behavioural responses which in turn influence the world. The perceptual model of the HGF formalises how belief states are formed on the three levels of uncertainty about the generation of the stimuli. The response model links this perception to the behavioural responses. The perceptual and response model are fitted in an iterative process to determine the most probable parameter values given sensory inputs and observed responses

We modelled participants’ reaction times using a three-level HGF [8, 18], a computational model of learning and adapting to changing information that formalises how the true hidden states governing the world are tracked as belief states based on the sensory input and the behavioural responses (see Fig. 3). The application of this model extracted participant-specific trial-by-trial belief trajectories shown as *x* in Fig. 3 as well as model parameters capturing how a person processes and reacts to different types of uncertainty, including tonic and phasic volatility. The HGF combines a perceptual model, which describes how the participant perceives information and forms the beliefs about hidden states, and a response model, which describes how these perceptions are translated into behavioural responses. These models are refined through an iterative optimisation process to determine the most probable parameter values given sensory inputs and observed responses. The perceptual model features three nested levels of beliefs: the level of the stimulus, the cue-outcome association as well as the environment. Predictions on the second and third level are governed by tonic volatility parameters *ω*_*2*_ and *ω*_*3*_ capturing the expected stability of the respective level, while the first level is influenced by the currently perceived stimulus. The response model links these beliefs to the response of the participant. Tonic environmental volatility estimates are captured by the perceptual model and represent a participant’s background expectation of change, while the impact of phasic volatility is estimated by the response model and represents the influence of dynamic and unexpected environmental uncertainty on responses.

We included two variations of the perceptual model, the original HGF (oHGF) and the enhanced HGF (eHGF), as implemented in the TAPAS toolbox v6.0.2 [40]. The oHGF is described in detail in Mathys et al. [8, 18] and the eHGF in Hess et al. [19]. For both, we employed a version for binary outcomes, modelling the association between cue and outcome (e.g., high tone followed by positive facial expression) and including trial-by-trial perceptual uncertainty (difficulty of the decision). We paired these perceptual models with the same response model based on reaction times used in Lawson et al. [9] which is shown in Eq. 1. Response model. Here, stimulus surprise captures the negative base-2 logarithm of the probability of observed outcome; thus, the more likely the observed outcome, the lower the surprise. Stimulus uncertainty refers to the predicted variance on the first level, while cue-outcome uncertainty is a measure of the variance on the second level. Phasic volatility assesses the environmental uncertainty.

1 $$\eqalign{ & logRT = {\beta _0} + {\beta _1} \cdot {\rm{stimulus}}\>{\rm{surprise}} \cr & + {\beta _2} \cdot {\rm{stimulus}}\>{\rm{uncertainty}} \cr & + {\beta _3} \cdot {\rm{cue}}\_{\rm{outcome}}\>{\rm{uncertainty}} \cr & + {\beta _4} \cdot {\rm{phasicvolatility}} + \zeta \cr} $$

To assess these models, we adapted the workflow presented by Hess and colleagues [19]. The workflow includes the extraction of empirical priors from a combination of our pilot data and the data shared from Lawson et al. [17], parameter recovery and model identifiability based on simulated data, posterior predictive checks and random-effect analysis Bayesian model comparison as implemented in the VBA toolbox [41]. Learning rates capture the precision weights on the prediction errors, thus adjusting the impact the prediction error has on updating. On the second level, this is defined as the inverse of the second level precision, while on the third level it is the ratio between the estimated precision on the second level and the actual precision on the third level [8]. We computed learning rate updates for the second and the third level by computing the difference in median learning rate between 72 trials of the stable phases and the volatile phase. To aid their interpretation, we present learning rate updates from the Bayes optimal perceptual parameters minimising the Shannon surprise for this task.

We compared the HGF to a simpler learning model, the Rescorla-Wagner (RW) model [42, 43]. While we computed empirical priors for the RW model as well, they led to unsuccessful parameter recovery based on simulated data (see S3). Thus, we present data from the RW model with default priors for which parameter recovery was successful. The comparison of the preregistered oHGF with the RW model and the eHGF was not preregistered.

### Analysis with Bayesian linear (mixed) models

HGF parameters and behavioural responses were analysed with Bayesian models as implemented in brms [44]. Priors were individually set for each model based on previous literature and data-naïve prior predictive checks [9, 17, 20]. Models were evaluated using prior predictive checks, simulation-based calibration and posterior predictive checks [20, 45, 46]. Maximal slopes for the group-level were added, where possible [47, 48]. In addition to our preregistered model regarding HGF parameters, we analysed the influence of all HGF parameters except base reaction times (RT) on group using a Bayesian linear model with a Bernoulli distribution. While we preregistered training a classifier to predict group, we did not specify the model and decided to use this model with a Bernoulli distribution for ease of interpretability. Details for all models can be found in the supplementary materials.

We assessed all hypotheses for credibility using the brms::hypothesis [44] function with a preregistered threshold of *ɑ* = 0.05, with one-sided testing for directional hypotheses. For explorative testing, we use the term “very strong evidence” for posterior probabilities above 97.5%, “strong” for above 95%, “moderate” for above 90%, “small” for above 80% and “anecdotal” for above 70%. Additionally, we used the region of practical equivalence (ROPE), capturing outcome values between ± 0.1 for standardised parameters, and the highest density interval (HDI) to assess evidence for equivalence [49]. An HDI completely inside the ROPE supports the null hypothesis. Where we used Bayesian ANOVAs, Bayes Factors (BF) are log-transformed so that negative values indicate evidence *against* and positive values evidence *in favour of* a specific model over the intercept-only model. We computed effect sizes *δ* by dividing the effect by the square root of the sum of all squared variances of the respective model [50].

## Results

### Reaction times, pupil sizes and accuracies

**Fig. 4:**
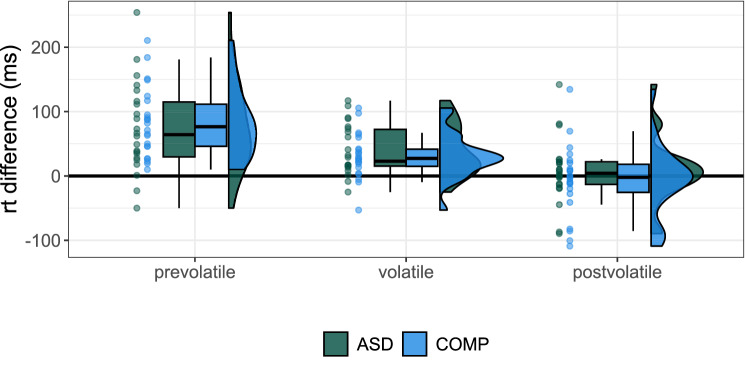
Difference in reaction times between expected and unexpected trials across the phases. While during the prevolatile and the volatile phase most participants reacted faster on expected trials, this effect seems to be lost by the postvolatile phase

There was no credible evidence for differences between autistic and comparison adults with regards to the effect of *Expectancy* (*estimate* = 0 [-0.01, 0.01], *posterior probability* = 36.57%, *δ* = -0.018 [-0.122, 0.087], inside ROPE = 92.7%, HDI = [-20.23, 11.1], see Fig. 4) or transition effects (prevolatile to volatile: *estimate* = 0 [-0.02, 0.02], *posterior probability* = 48.31%, *δ* = 0.005 [-0.155, 0.167], inside ROPE = 81.47%, HDI = [-26.02, 22.77]; volatile to postvolatile: *estimate* = 0 [-0.04, 0.04], *posterior probability* = 54.78%, *δ* = -0.009 [-0.177, 0.157], inside ROPE = 82.92%, HDI = [-23.02, 25.14]) based on reaction times. Similarly, there was no credible difference in the effect of *Expectancy* on pupil sizes in autistic compared to non-autistic adults (*estimate* = 0 [0, 0.01], *posterior probability* = 22.16%, *δ* = -0.037 [-0.133, 0.06]), in contrast to our hypothesis. Across groups, there was very strong evidence that expected trials led to faster responses than unexpected trials (*estimate* = -0.07 [-0.1, -0.05], *posterior probability* = 100%, *δ* = -0.245 [-0.329, -0.162]) and easy trials led to faster responses than hard trials (*estimate* = -0.07 [-0.09, -0.04], *posterior probability* = 100%, *δ* = -0.222 [-0.318, -0.126]), suggesting that our task manipulations were successful.

Accuracies were generally high, with a grand average of 90.8% accurate responses across diagnostic groups. An explorative Bayesian ANOVA of the ranked accuracies revealed influences of *Group*, *Expectancy* and *Difficulty* but no interaction effects (log(*BF*) = 41.15). Accuracies were higher in the comparison than the autistic group (ASD: 88.7 ± 14.2%; COMP: 92.9 ± 11.1%) and in the expected than the unexpected trials (expected: 92.3 ± 9.2%; unexpected: 89.3 ± 15.7%). Furthermore, accuracies were lower in the hard condition (easy: 93.7 ± 11.5%; hard: 86 ± 14.1%).

### Model evaluation

We simulated data based on empirical priors for the oHGF and the eHGF as well as based on default priors for the RW. We used this data to assess parameter recovery as well as model identifiability. Parameter recovery revealed significant correlations between simulated and recovered parameters for all three models (eHGF: *r*_*environmental tonic volatility*_ = 0.72, *r*_*phasic volatility*_ = 0.75; oHGF: *r*_*environmental tonic volatility*_ = 0.66, *r*_*phasic volatility*_ = 0.85; RW: *r*_*α*_ = 0.48; for all other value consult S3). Model identifiability revealed a balanced accuracy of 74% across all three models. While the RW model was correctly identified as the model based on which the data was simulated in 99% of the cases, the oHGF was correctly identified in 73% and the eHGF in 50% of the time. These models were confused with each other and not with the RW, showing that the models are very similar (see Figure S5).

**Fig. 5:**
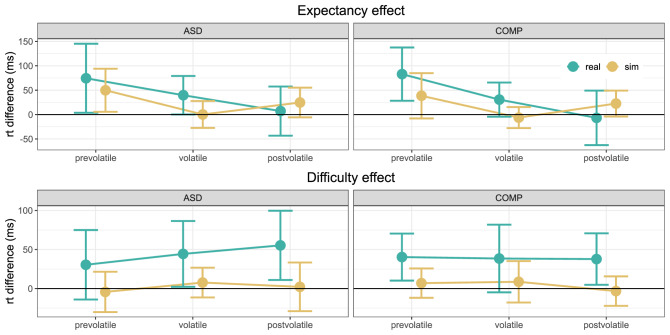
Posterior predictive checks comparing the reaction times of the participants to those simulated based on the oHGF. While the expectancy effect observed in the participant data is largely reproduced in the simulated reaction times, the difficulty effect capturing the difference in responses to hard and easy stimuli is not reproduced by the model. Error bars show standard deviations

After these checks, we fit the real data with all three models. Model comparison based on the real data revealed the oHGF as the best fitting model (eHGF: *estimated model frequency* = 37.62%, *exceedance probability* = 5.19%; oHGF: *estimated model frequency* = 61.35%, *exceedance probability* = 94.81%; RW: *estimated model frequency* = 1.03%, *exceedance probability* = 0%). Visual posterior predictive checks revealed that this model captured the *Expectancy* but not the *Difficulty* effect (see Fig. 5 and S3). For a formal evaluation of the posterior fit, we performed two checks: first, we correlated the median reaction times for the conditions for each participant based on the observed and the simulated data to capture their similarity. Second, we refitted the reaction time model to the simulated reaction times to compute the overlap of the posterior distributions of the parameter estimations. The within-condition correlations between the median of the observed and the simulated data were strong, ranging from *r* = 0.58 [0.37, 0.75] for the medium difficulty, unexpected trials in the volatile phase to 0.94 [0.90, 0.97] for the easy, expected trials in the volatile phase (for all values see Table S1). These correlations suggest comparable behavioural patterns in the simulated and the observed data. Furthermore, running the behavioural model on the simulated data revealed a wide range of overlap between the estimates based on this model and the estimates based on the model of the observed data (mean = 54.46 [2.78, 90.04]). However, overlap values are higher for parameter estimates regarding the predictor group and interactions with group (mean = 63.29 [38.54, 91.42], for all values see Table S3). Since our focus is on the *Expectancy* as a result of the probabilistic learning processes, we judge this to be an acceptable posterior predictive fit.

Furthermore, log model evidence (LME) was comparable between both groups, suggesting that the model fit participants from both groups equally well (log(*BF*) = -1.045). Thus, we extract participant-specific belief states and their trajectories from the oHGF model for further analysis.

### Volatility parameters and learning rate updates

**Fig. 6:**
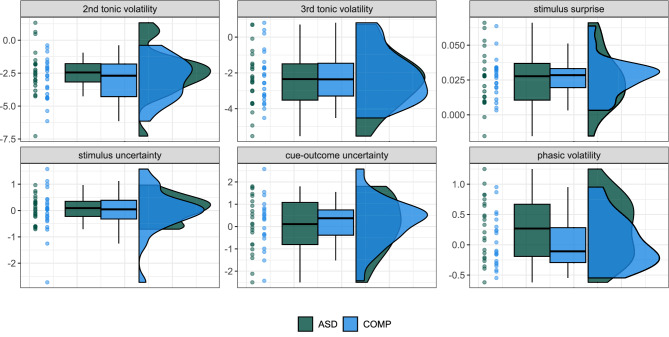
Participant-specific HGF parameters extracted from the perceptual (2nd and 3rd level tonic volatility) and the response model. The dots show individual participant parameters, while the boxplot and distribution graphs summarise all participants in a group. Boxplot hinges show the first and third quartiles with the bold line denoting the median. Whiskers extend ± 1.5 times the interquartile range. While tonic volatility parameters show little difference between groups, phasic volatility estimates are higher in the autistic than the comparison group

There was no credible difference between the influence of phasic volatility in autistic and comparison adults (*estimate* = -0.1 [-0.22, 0.01], *posterior probability* = 92.54%, *δ* = -0.427 [-1.015, 0.141], see Fig. 6), contrasting our hypothesis and previous findings. However, there was moderate evidence in favour of a difference with the posterior probability approaching the preregistered cutoff of 95%, falling slightly short of credible evidence but also not lending support to comparable phasic volatility with only 20.59% of posterior estimates inside the ROPE (HDI = [-0.03, 0.24]). Similarly, strong evidence pointed towards increased influence of phasic volatility predicting ASD in the explorative Bernoulli model, with a posterior probability of 97.17% (*estimate* = 0.81 [-0.02, 1.68], inside ROPE = 4.93%, HDI = [-0.02, 1.68]). Last, there was no evidence towards higher influence of environmental tonic volatility on performance in autistic than comparison adults (*estimate* = 0.03 [-0.24, 0.31], *posterior probability* = 41.61%, *δ* = 0.047 [-0.394, 0.499], inside ROPE = 68.79%, HDI = [-0.36, 0.29]).

**Fig. 7:**
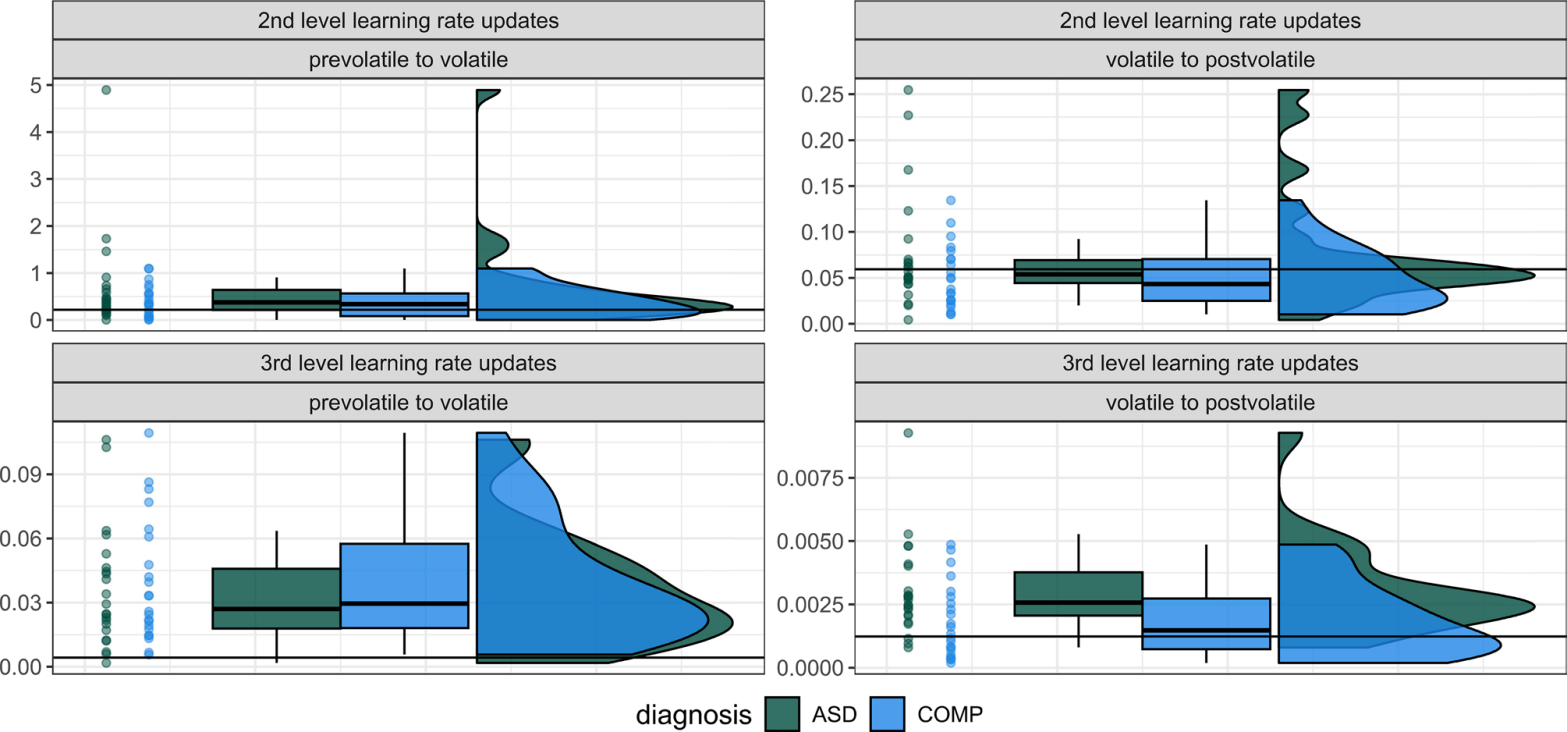
Learning rate updates on the second and the third level, capturing the absolute change from prevolatile to volatile and volatile to postvolatile phase. Learning rate updates were higher for the switch from prevolatile to volatile phase than for the switch from volatile to postvolatile as well as for learning about cue-outcome associations, captured by the learning rate on the second level, than learning about the environment, captured by the learning rate on the third level. Black, horizontal lines show the learning rate updates based on the Bayes optimal perceptual parameters. Participants in both groups showed stronger updates from the prevolatile to the volatile phase on the environmental level, while only autistic participants additionally showed increased updates on the environmental level moving from the volatile to the postvolatile phase

For the model regarding learning rate updates, posterior predictive checks revealed a suboptimal fit of estimated parameters for the change from volatile to postvolatile phase. Thus, we complemented the preregistered model with an explorative Bayesian ANOVA of the ranked learning rates. Posterior distributions of the preregistered model showed small but not credible evidence for increased learning rate of environmental changes (*estimate* = 0.14 [-0.07, 0.35], *posterior probability* = 87.01%, *δ* = 0.266 [-0.205, 0.737]) and small evidence *against* the hypothesis of decreased learning rate for cue-outcome associations (*estimate* = 0.2 [-0.08, 0.47], *posterior probability* = 11.89%, *δ* = 0.376 [-0.257, 1.011], see Fig. 7). The Bayesian ANOVA revealed almost identical fit for two models, both containing the predictors *Level* and *Change* with one of them also including their interaction (without interaction log(*BF*) = 105.31, with interaction log(*BF*) = 105.27). However, there was only anecdotal evidence in favour of these two models compared to the model with the third-best fit which additionally included the predictor *Group* (log(*BF*) = 104.71). Thus, all results regarding the learning rate updates should be interpreted cautiously. For a visualisation of the learning rates used to compute the learning rate updates, please see Figure S11.

Recent studies on flexibility in emotional tasks as well as prediction errors elicited by faces in autism revealed sex differences [51, 52]. Since our autistic sample involved 50% women and the sample in Lawson et al. [9]involved only 25% women, we visually and statistically explored gender effects which revealed neither effects of gender nor credible interactions between gender and group (see S9).

## Discussion

Bayesian Brain theories have proposed modified predictive processing as a core mechanism underlying ASD. Lawson and colleagues [9] advanced this hypothesis through an elegant task design that manipulated expectancy and volatility during probabilistic learning. In the current study, we assessed whether their findings of increased tonic and phasic volatility estimates paired with learning rate update modifications in autistic adults extend to a core challenge for autistic people: facial emotion recognition [12]. Using a Bayesian workflow, the current results revealed no credible differences between autistic and comparison adults in our preregistered models, neither through HGF extraction nor through conventional analyses of reaction times and pupil sizes. The perceptual model of the HGF yielded no evidence for differences in tonic volatility estimates between groups, rejecting our initial hypothesis based on the results of Lawson and colleagues [9]. The response model produced moderate to strong evidence towards increased phasic volatility estimates in autistic adults, though the evidence fell short of our preregistered threshold for credibility. In the following, we discuss these findings in the context of other studies using the HGF and recent findings regarding prior integration and intolerance for uncertainty in ASD.

### What is learned: from visual categories to socio-emotional states

The current task had the same trial structure and underlying probabilities as the task used in Lawson et al. [9]. However, both the stimuli and the decision differed between the two studies. Where Lawson and colleagues used house and face stimuli asking participants to decide which category of stimulus was presented, we chose to use only face stimuli which were varying in emotional valence. Participants were asked to determine whether the facial expression of the faces were associated with positive or negative emotions. We adjusted the Lawson paradigm to this socio-emotional context based on the core symptoms of autism [7], hoping to tie differences in predictive processes to common deficits in emotion recognition in autism [12, 33]. However, the current data provide less robust evidence for differences between autistic and non-autistic adults in probabilistic associative learning in a socio-emotional context with some evidence for differences in HGF parameters but no evidence for differences on reaction times, accuracies or pupil sizes. This pattern of results indicates that the original findings by Lawson et al. [9] are less robust than previously assumed, even in the context of socio-emotional stimuli which arguably should even increase group differences between autistic and non-autistic adults. While the task may lack sensitivity in the socio-emotional domain because the valence decision was too easy, this would have made the current task functionally even more similar to the original house/face categorisation. Thus, we would still expect a similar pattern as in the original findings, which is not supported by the current results. In the following paragraphs, we individually discuss the findings regarding phasic and tonic volatility as well as learning rate updates in more detail.

### Tonic volatility: accumulating evidence for similarity

The current findings strengthen the emerging evidence for comparable tonic volatility estimates in autistic and non-autistic adults. Multiple studies using the HGF have now documented this similarity: probabilistic associative learning tasks [13, 14], the Wisconsin Card-Sorting task measuring cognitive flexibility and set-shifting [15] and a size-illusion task [16], experiment [9]. Nonetheless, some evidence for group differences does exist [9], [16], experiment [1], and these studies also found differences in learning rates or their updates, while others found comparable learning rates in autistic and non-autistic adults [15], [16], experiment [9]. The mixed results regarding tonic volatility differences are especially puzzling, since there is no clear pattern emerging regarding possible task specifications driving the differences. In fact, studies using the same computational models based on data from similar tasks have produced both evidence for and against differences, for example the current study and Lawson et al. [9]. The difference in results between the current study and Lawson et al. [9] could be due to the varying learning context (socio-emotional versus non-social stimuli), but based on the symptom profile of ASD, it is unlikely that the socio-emotional context reduces differences between autistic and non-autistic adults. Thus, the current corpus of literature does not support a general modification of tonic volatility in autism, suggesting that generalised precision regulation accounts seem to be less robustly replicable than previously thought.

### Phasic volatility: suggestive but inconclusive evidence

Phasic volatility estimates differed between autistic and non-autistic adults, with a posterior probability of 92.5% — just below our 95% threshold. This evidence pointed towards increased influence of phasic volatility in autistic compared to non-autistic adults, possibly supporting Lawson and colleagues’ [9] results. Similar to the current findings, Sapey-Triomphe and colleagues reported a comparable perception of tonic volatility combined with altered response model parameters [13] and modified neural correlates of prediction [14] in a probabilistic associative learning task. These converging findings suggest that autistic adults may not differ from non-autistic adults in perceiving the baseline volatility of the environment, but rather in the reaction to sudden environmental change. This interpretation would also fit with the elevated intolerance of uncertainty consistently reported in ASD [53]. In a recent study using a duration reproduction task, autistic adults showed a tendency to specifically overweight sensory information in belief updating, instead of in the posterior, leading to a high rate of belief updating [5]. This mechanism may explain why tonic volatility did not show any differences between autistic and non-autistic adults in the current study, but phasic volatility capturing trial-by-trial prediction of behaviour did. However, because the current evidence for increased phasic volatility estimates in autistic adults fell short of the preregistered threshold, we urge caution until replication confirms these results.

### Learning rate updates: mixed results

We observed small but non-credible evidence for *increased* learning rate updates following environmental changes in ASD. This finding resembles Lawson et al.’s [9] finding of *increased* learning rate updates for environmental changes, though they also found *decreased* learning rate updates for cue-outcome associations. In contrast, we found small evidence of *increased* learning rate updates for cue-outcome associations. Small differences in the construction of the phases between both studies could have contributed to these differences. In the current task, there was a prevolatile phase where cue-outcome associations were stable for 72 trials, followed by a volatile phase where these associations were flipped multiple times, and finally a postvolatile phase where the opposite cue-outcome associations to the prevolatile phase were stable for 72 trials. We used the first and last 72 trials of the volatile phase to compute learning rate updates from the respective stable phases. While the structure in Lawson et al. [9] was similar, the second stable phase was again followed by a second volatile phase and the authors computed learning rate updates based on these last two phases only. These inconsistent patterns across studies challenge the robustness of proposed differences in learning rate updates between autistic and non-autistic adults.

### Hypo-priors in autism

Our null results regarding volatility perception align with a broader pattern of mixed evidence across Bayesian accounts of autism. An early and influential theory proposed that autistic individuals form weaker priors, so-called hypo-priors, leading sensory input to dominate perception and cognition [4]. Yet a growing corpus of studies have failed to find the systematic perceptual differences this account predicts. Using a tactile learning task, Sapey-Triomphe and colleagues [54] found no differences in prior adjustment between autistic and non-autistic adults but did observe context-dependent adaptation differences — pointing to altered learning rather than altered perception. Similarly, Liu et al.[55] found that autistic and non-autistic adolescents showed comparable prior uncertainty in a social trust game, even though their prior beliefs about a partner’s trustworthiness differed. A converging picture emerges across these studies and the current study: perception of uncertainty and the formation of priors appear largely intact in autism, while differences arise in how these representations are updated and translated into behaviour. In the current results, the perceptual model yielded no group differences in tonic volatility, whereas the response model produced moderate evidence for increased phasic volatility in autistic adults — a dissociation between perception and its behavioural expression that mirrors the pattern reported by Sapey-Triomphe and colleagues [13, 54]. Thus, altered use and adaptation of priors, rather than weakened priors themselves, may be the more promising mechanism for understanding predictive processing differences in autism.

### Model fit and alternative computational frameworks

The current study assessed whether reported differences in HGF-extracted parameters of predictive processes between autistic and non-autistic extend to a socio-emotional context; while we compared model fit to a simpler learning model, an exhaustive evaluation of the full range of potentially relevant computational models of prediction in autism was not the aim of the current study. While the model passed several validation checks, posterior predictive checks showed an incomplete capture of expectancy and especially difficulty effects on reaction times. This suboptimal fit suggests that alternative computational models may better fit the task-specific data than the specific HGF used in the current study. While one possibility would be to develop a response model for the HGF to increase model fit, other computational approaches like Kalman filters may capture autism-related differences better. For example, a recent study by Shi et al. [5] investigated prior updating in ASD using the central tendency effect in combination with Kalman. They found an initial underweighting of priors in autistic adults, resonating with some previous findings indicating atypical priors [13, 56], which normalised by the end of the task, resonating with contrasting previous findings of intact priors in ASD [57]. This approach showed that careful investigation of the full time-course of performance can potentially resolve discrepant findings. A similar mechanism might have been at play in the current task without being captured by the HGF architecture.

### Reaction times and pupil sizes: comparable expectancy effect

Mirroring the similar HGF estimates between autistic and comparison adults, we found comparable transition effects on reaction times and comparable expectancy effects on both reaction times and pupil sizes, contrasting with Lawson et al.’s [9] results. Following their approach, we examined associations between precision-weighted prediction error and expectancy within the specific time windows for which they reported group differences. Again, the current data failed to support previous findings. Therefore, we conclude that probabilistic associative learning and predictive processes are largely comparable between autistic and comparison adults.

### Limitations

Certain limitations warrant consideration when interpreting the current results. First, while we reached the preregistered sample size based on a power analysis to detect a medium-sized effect, the performed analysis differed from the power analysis. Thus, our group sizes may have been insufficient to detect robust but subtle differences between autistic and comparison adults. While our sample was comparable to the sample in Lawson et al. [9] in size (44 participants in our sample, 49 in Lawson et al.), age (our sample: *mean* = 29.9; Lawson et al.: *mean* = 35.5), IQ (our sample: *mean* = 114; Lawson et al.: *mean* = 117) and ASD diagnoses (about 80% Asperger syndrome in both samples), there was a difference in gender distribution. In light of evidence for sex differences in flexibility in emotional tasks and prediction errors of faces in autism [51, 52], this difference in sample composition may have contributed to the differences in results. While explorations did not reveal any gender differences in the current data, the sample size is too small for strong conclusions on this dimension. Second, visual inspection of the reaction time data revealed that participants first learned the associations in the prevolatile phase, but by the postvolatile phase, expected and unexpected trials elicited comparable reaction times. Increasing the number of trials within each block of stable cue-outcome associations might have allowed our participants to form stronger associations, leading to differences between expected and unexpected trials in all phases of the task. Last, we chose to limit the emotion recognition in the current task to a simple valence decision (positive or negative emotion). While this allowed us to maximise the number of trials which were included in the analysis and modelling of the reaction times, the resulting ceiling effect limits the interpretability of the accuracies which are high throughout the task. Thus, researchers of future studies may consider adjusting the task design to fit their needs.

## Conclusions

While some theories suggest differences in predictive processing underlie ASD, the current findings challenge the robustness of empirical evidence for differences in volatility estimates and learning rate updates between autistic and non-autistic adults. The current study failed to find previously reported HGF-extracted differences using a task that specifically targeted a core deficit of ASD – facial emotion recognition. This null result raises questions about the generalisability and clinical relevance of previous findings. Alterations in the way priors are iteratively updated [5, 58] may hold greater promise for understanding autism-related differences in predictive processing.

## Supplementary Information

Below is the link to the electronic supplementary material.

Supplementary Material 1

## Data Availability

The datasets generated and analysed during the current study are available on OSF, [https://osf.io/krp4s].

## Abbreviations

ADHD: Attention-Deficit/Hyperactivity Disorder
ASD: Autism Spectrum Disorder
BDI: Beck Depression Inventory
BF: Bayes Factor
HDI: Highest Density Interval
HGF: Hierarchical Gaussian Filter
IQ: intelligence Quotient
KDEF: Karolinska Directed Emotional Faces
LME: Log Model Evidence
LMU: Ludwig Maximilian University
RADS-R: Ritvo Autism Diagnostic Scale-Revised
ROPE: Region of Practical Equivalence
RT: Reaction Times
