# Supplementary material for: Predicting emotional valence in autism: a preregistered study in the Bayesian Brain framework
Source: Mol Autism. 2026 Jul 21;17:32. doi: 10.1186/s13229-026-00730-3 (PMC13435570; doi:10.1186/s13229-026-00730-3)

Supplementary materials

Predicting emotional valence in autism: a preregistered study in the Bayesian Brain framework

Plank et al.

2026-04-25

S1 Package versions

The following packages are used in this RMarkdown file:

```
## [1] "R version 4.5.2 (2025-10-31)"

## [1] "knitr version 1.51"
## [1] "ggplot2 version 4.0.2"
## [1] "brms version 2.23.0"
## [1] "designr version 0.1.13"
## [1] "bridgesampling version 1.2.1"
## [1] "tidyverse version 2.0.0"
## [1] "ggpubr version 0.6.2"
## [1] "ggpattern version 1.2.1"
## [1] "correlation version 0.8.8"
## [1] "ggrain version 0.1.0"
## [1] "bayesplot version 1.15.0"
## [1] "SBC version 0.5.0.9000"
## [1] "rstatix version 0.7.3"
## [1] "BayesFactor version 0.9.12.4.7"
## [1] "effectsize version 1.0.1"
## [1] "bayestestR version 0.17.0"
```

S2 Methods

S2.1 Preprocessing of pupil sizes

We classified any extreme values (above 8 and below 2mm) as well as participant-specific extreme velocities and blinks as artefacts. Then, we low-pass filtered the data with a threshold of 4Hz based on Kret and Sjak-Shie (2019), smoothed the data and interpolated all artefacts shorter than half a second with cubic spline interpolation. Next, data was downsampled to 100Hz and trials with more than one third of the datapoints missing were excluded from analysis. Last, we applied correction based on a baseline of 200ms before onset of the face presentation. We visualised data quality and excluded trials if the pupil trace indicates recording artefacts or baseline artefacts as well as if the baseline pupil size is considered an outlier (absolute z-score larger than 2).

S2.2 Bayesian linear (mixed) models

All contrasts are set to sum coding. The models for the volatilities extracted from the HGF and for the pupil sizes used a Gaussian likelihood, while both the reaction times and learning rate updates were modelled using a lognormal distribution. The reaction time model additionally included a shift. All models included the predictor Group (ASD, COMP). The reaction time model also included Expectancy, Phase and Difficulty as well as all interactions on the population-level, and participant with slopes for Expectancy, Phase, Difficulty and their interactions as well as trials with slopes for Group on the group-level. The hypothesis regarding pupil sizes was evaluated using a model with the predictors Group, Expectancy and their interaction as well as intercepts for the participants on the group-level. Since pupil sizes are sensitive to physical movements (Mathôt & Vilotijević, 2023), we include RT of each trial instead of including trial on the group-level, as preregistered. The model for the learning rate updates included Group, Level (environmental or cue-outcome association) and Change (stable to volatile or volatile to stable) on the population-level as well as participants with slopes for Level and Change on the group-level.

S3 Model evaluation

S3.1 Parameter recovery and model identifiability

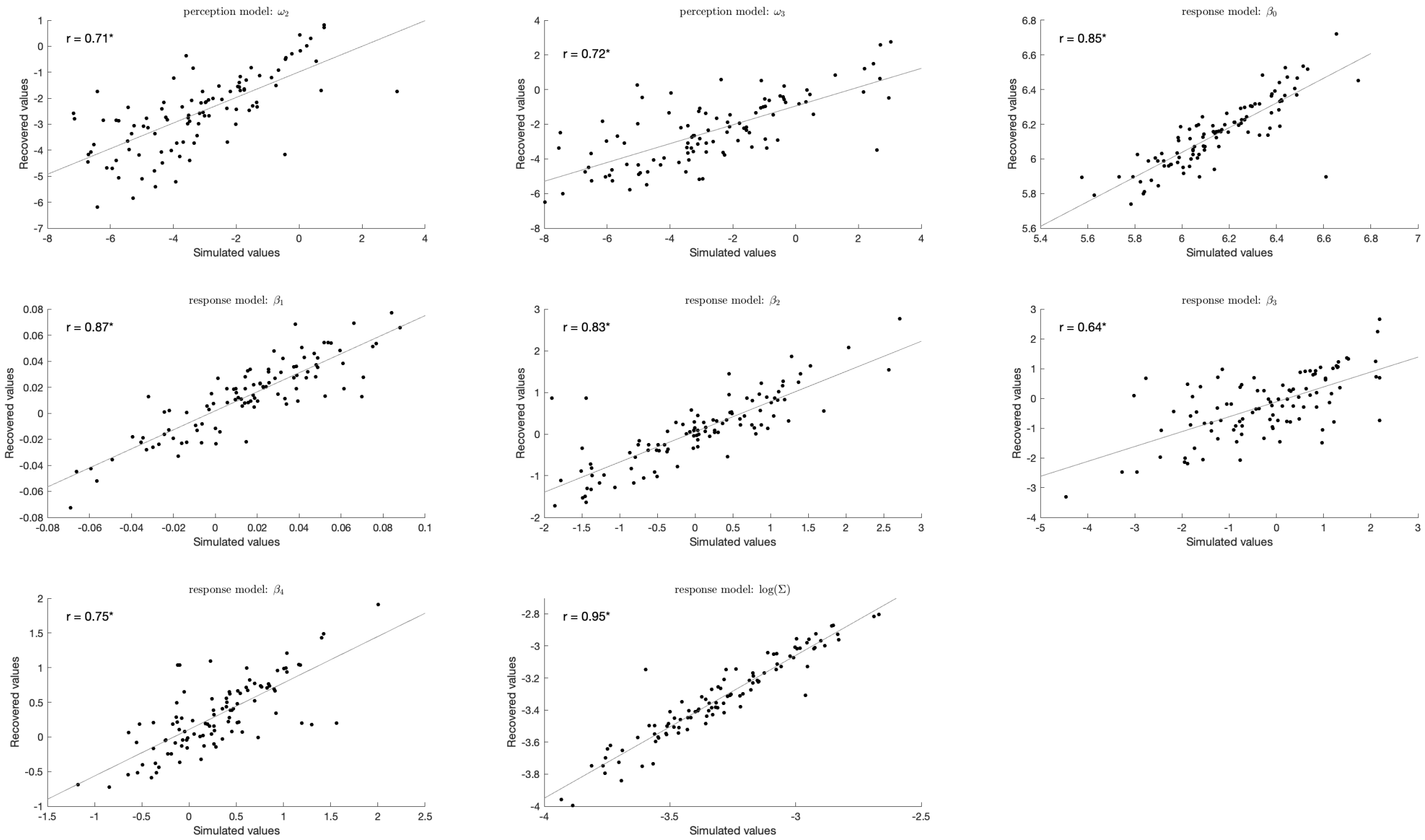

Figure S1: Enhanced HGF with empirical priors

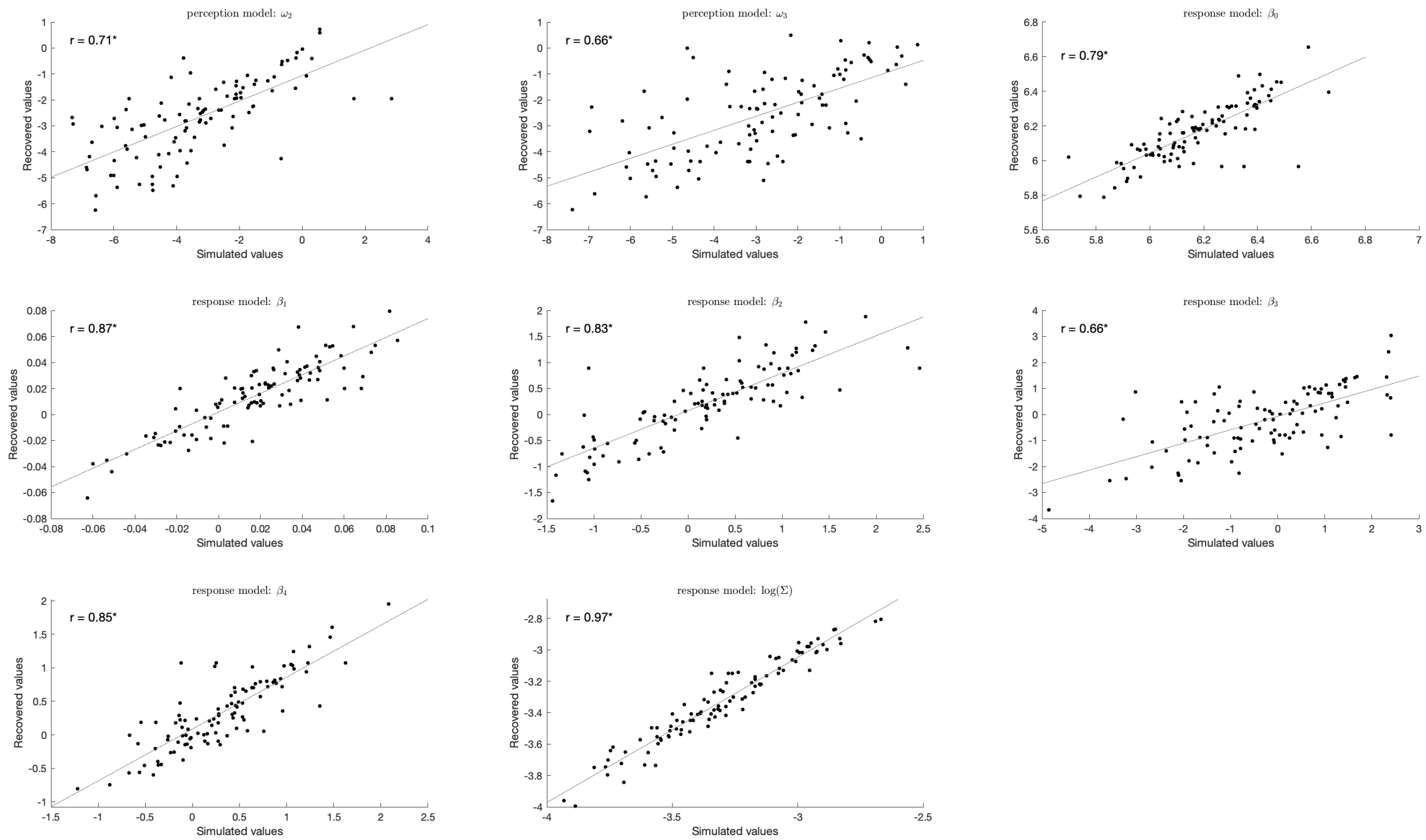

Figure S2: Original HGF with empirical priors

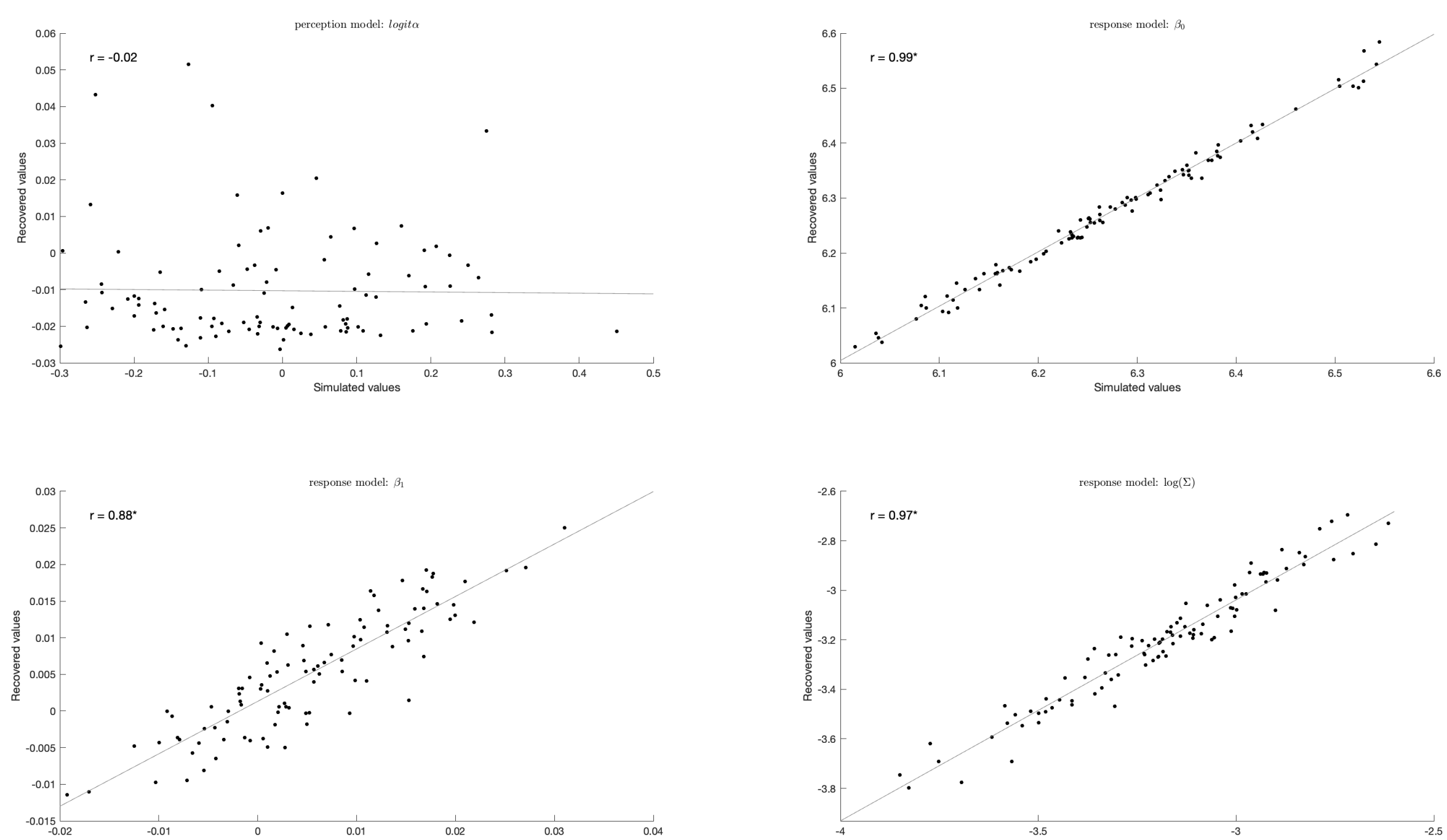

Figure S3: Rescorla-Wagner with empirical priors

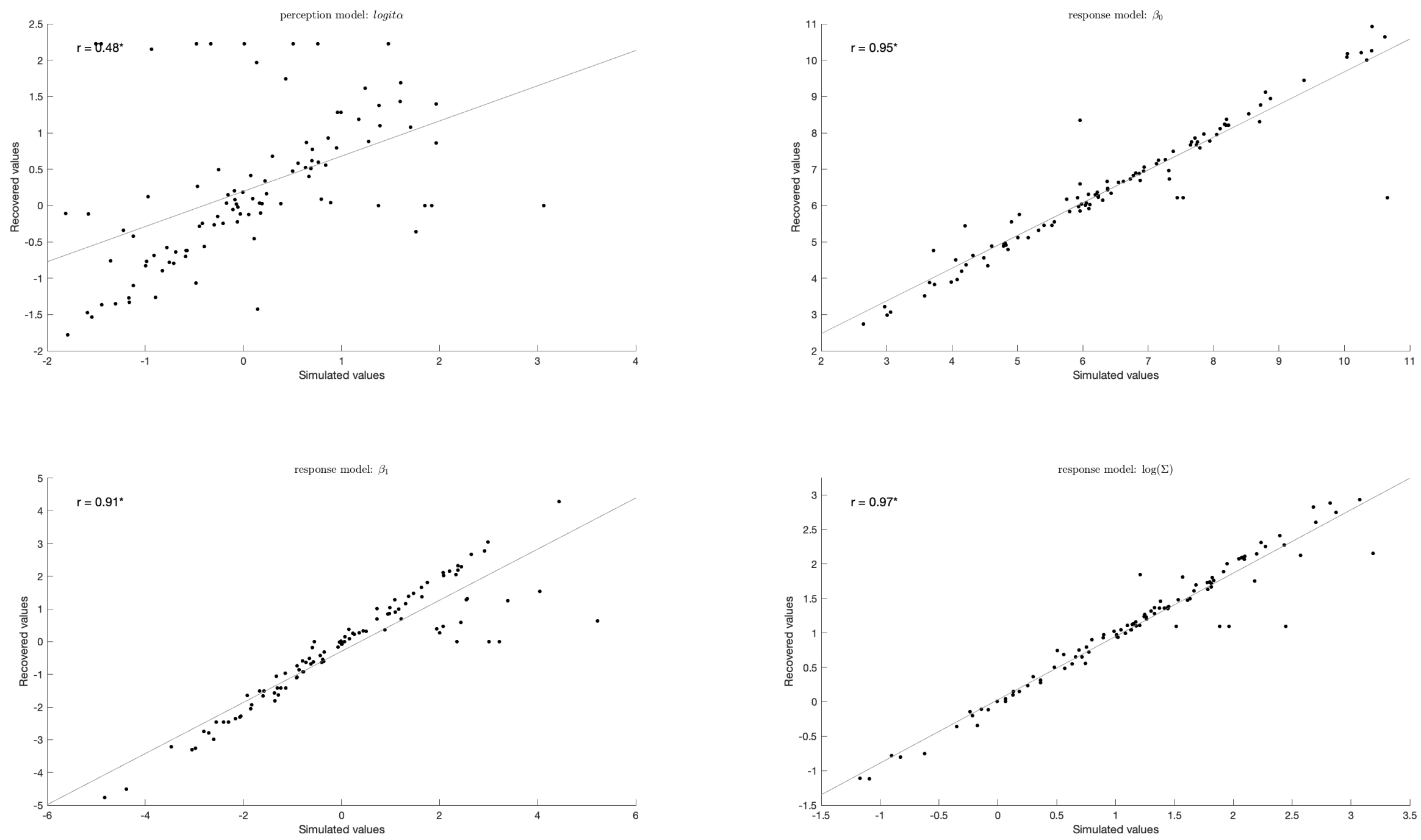

Figure S4: Rescorla-Wagner with default priors

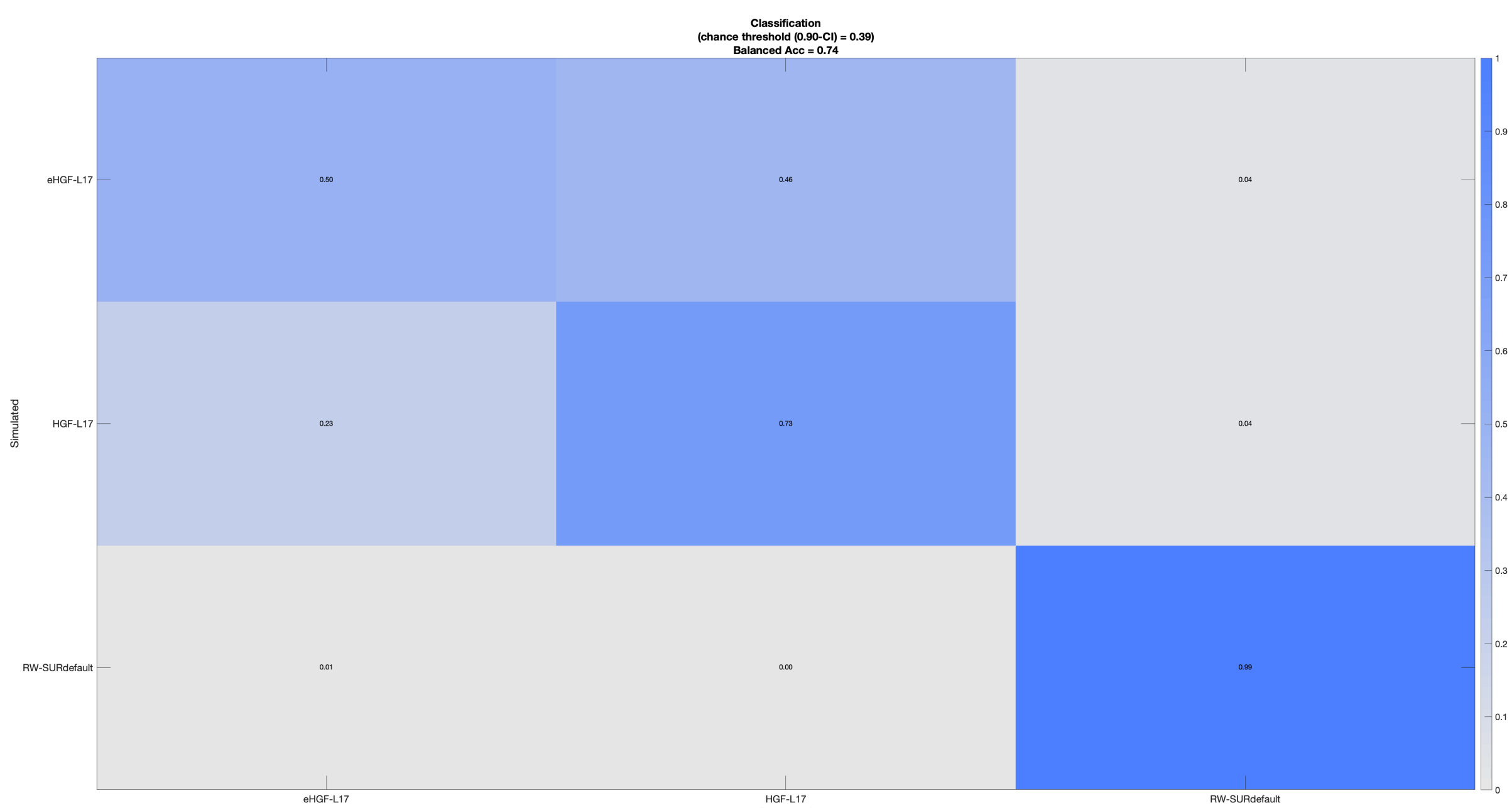

Figure S5: Model identifiability

S3.2 Posterior predictive checks for oHGF

Here, we compare simulated and observed reaction times. The simulated reaction times were simulated based on the model which best fit the data, namely the original HGF (oHGF).

Table S1: Within-condition correlations between median values of simulated and observed reaction times

Condition	rho	CI_low	CI_high	pd	BF	n_Obs
volatile - expected - easy	0.936	0.898	0.965	1	2.083329e+18	44
volatile - expected - medium	0.904	0.833	0.947	1	9.580647e+14	44
prevolatile - expected - hard	0.896	0.835	0.951	1	1.587879e+14	44
postvolatile - expected - medium	0.890	0.812	0.939	1	9.217609e+13	44
postvolatile - expected - easy	0.890	0.817	0.942	1	8.381426e+13	44
prevolatile - expected - medium	0.876	0.791	0.927	1	7.588768e+12	44
prevolatile - expected - easy	0.863	0.749	0.935	1	1.457185e+12	44
volatile - expected - hard	0.862	0.778	0.930	1	1.587908e+12	44
postvolatile - expected - hard	0.855	0.754	0.920	1	5.547958e+11	44
volatile - unexpected - easy	0.757	0.619	0.859	1	5.393531e+07	44
postvolatile - unexpected - easy	0.745	0.601	0.864	1	2.665158e+07	44
prevolatile - unexpected - medium	0.744	0.620	0.859	1	2.236104e+07	44
prevolatile - unexpected - hard	0.738	0.591	0.849	1	1.654123e+07	44
prevolatile - unexpected - easy	0.683	0.503	0.815	1	5.793616e+05	44
volatile - unexpected - hard	0.679	0.501	0.810	1	3.980486e+05	44
postvolatile - unexpected - medium	0.670	0.486	0.804	1	2.616542e+05	44
postvolatile - unexpected - hard	0.617	0.434	0.777	1	2.473718e+04	44
volatile - unexpected - medium	0.583	0.367	0.749	1	5.075359e+03	44

Table S2: Within-condition mean and SDs of observed and simulated RTs

phase	expected	difficulty	mean observed	mean simulated	SD observed	SD simulated
prevolatile	expected	easy	544.2	594.4	78.6	93.1
prevolatile	expected	medium	575.5	593.4	79.0	84.7
prevolatile	expected	hard	587.7	598.5	88.7	94.5
prevolatile	unexpected	easy	648.8	649.5	138.3	120.2
prevolatile	unexpected	medium	650.9	640.4	116.8	119.4
prevolatile	unexpected	hard	644.6	631.3	125.2	105.7
volatile	expected	easy	546.2	598.5	74.1	88.3
volatile	expected	medium	553.0	603.2	73.8	92.4
volatile	expected	hard	590.7	608.9	83.9	87.6
volatile	unexpected	easy	598.4	610.4	84.4	90.2
volatile	unexpected	medium	584.8	596.8	91.6	80.0
volatile	unexpected	hard	609.5	600.7	107.0	102.7
postvolatile	expected	easy	552.9	593.6	80.1	87.7
postvolatile	expected	medium	566.2	587.2	74.5	83.2
postvolatile	expected	hard	605.1	591.3	100.0	92.0
postvolatile	unexpected	easy	570.6	607.0	123.9	92.6
postvolatile	unexpected	medium	565.0	617.6	113.0	96.0
postvolatile	unexpected	hard	595.5	614.7	100.7	97.2

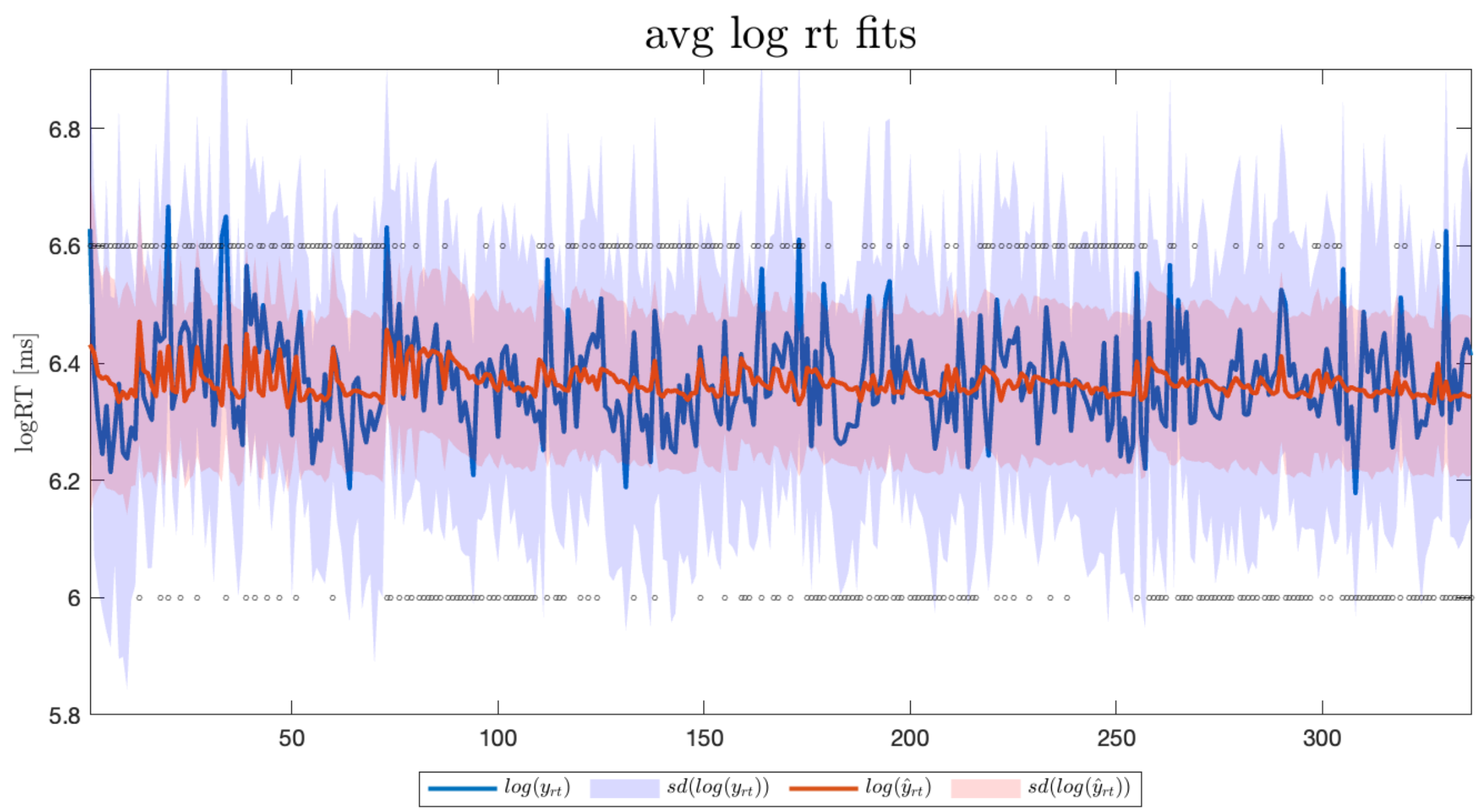

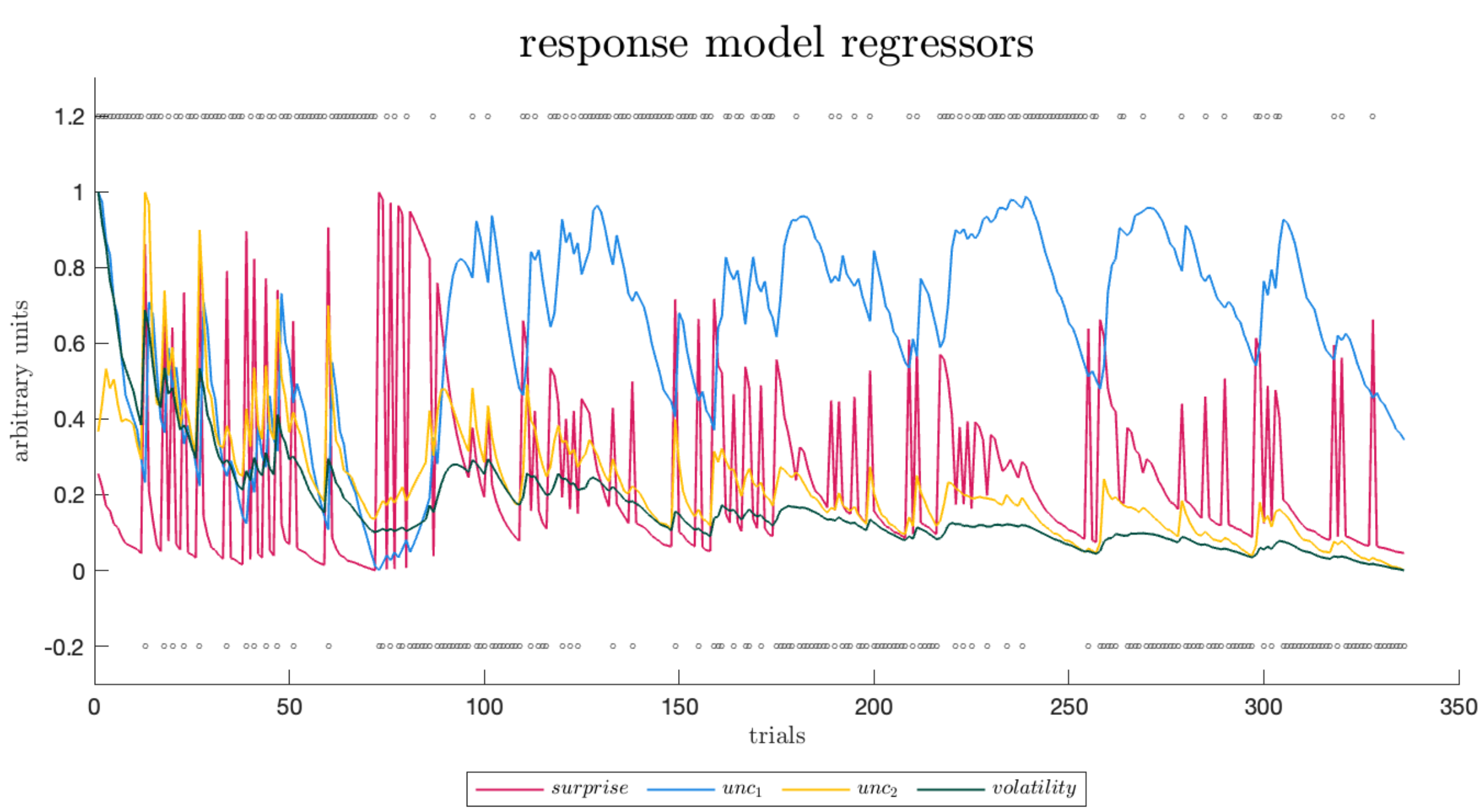

Figure S6: Task development of average log(rt) and simulated log(rt) as well as response model parameters

Table S3: Posterior predictive checks: comparison of model based on simulated and model based on observed data

effect	overlap	estimate based on observed RT	estimate based on simulated RT
b__diagnosis1	0.9279	0.0304	0.0308
b__diagnosis1:expected1	0.8955	-0.0013	-0.0022
b__diagnosis1:expected1:phase2	0.8023	0.0002	0.0004
b__diagnosis1:phase2	0.7896	-0.0007	-0.0028
b__diagnosis1:difficulty1	0.7698	0.0008	0.0014
b__diagnosis1:phase1	0.7657	0.0000	0.0061
b__diagnosis1:expected1:difficulty1	0.7484	-0.0013	0.0004
b__phase2	0.7445	-0.0071	-0.0077
b__phase1	0.6953	0.0269	0.0177
b__diagnosis1:phase2:difficulty2	0.6822	0.0059	0.0028
b__diagnosis1:phase2:difficulty1	0.6646	-0.0025	0.0009
b__expected1:phase1:difficulty1	0.6469	-0.0078	-0.0041
b__diagnosis1:expected1:phase1:difficulty2	0.6183	-0.0005	-0.0053
b__diagnosis1:expected1:phase1:difficulty1	0.6142	0.0034	0.0082
b__diagnosis1:expected1:phase2:difficulty1	0.6136	0.0010	-0.0033
b__expected1:difficulty2	0.6099	0.0020	-0.0011
b__expected1:phase1:difficulty2	0.6069	0.0075	0.0022
b__phase1:difficulty1	0.5789	-0.0022	0.0049
b__phase2:difficulty2	0.5421	-0.0110	-0.0041
b__phase2:difficulty1	0.5313	0.0077	0.0002
b__difficulty2	0.5261	-0.0086	-0.0025
b__expected1:phase2:difficulty1	0.5175	-0.0002	-0.0081
b__expected1:phase2:difficulty2	0.5034	-0.0037	0.0048
b__diagnosis1:difficulty2	0.4636	-0.0040	0.0011
b__diagnosis1:expected1:phase2:difficulty2	0.4329	-0.0047	0.0024
b__diagnosis1:expected1:difficulty2	0.4211	0.0025	-0.0034
b__diagnosis1:phase1:difficulty2	0.4066	-0.0067	0.0019
b__diagnosis1:expected1:phase1	0.4048	0.0052	-0.0024
b__expected1:difficulty1	0.3869	-0.0146	-0.0050
b__diagnosis1:phase1:difficulty1	0.3710	0.0062	-0.0033
b__phase1:difficulty2	0.3059	0.0174	0.0008
b__expected1:phase1	0.2467	-0.0374	-0.0222
b__expected1	0.1858	-0.0361	-0.0228
b__expected1:phase2	0.0314	0.0013	0.0245
b__difficulty1	0.0076	-0.0284	0.0036

S3.3 Comparison of LME across groups

```
## ---
##  Model:
## Type: BFlinearModel, JZS
## Intercept only
## Data types:
## diagnosis :  fixed
```

	bf	error	time	code
diagnosis	-1.04496	5.81e-05	Wed Oct 15 12:08:23 2025	144d96dd874c4

S4 Volatility parameters and learning rate updates

S4.1 Phasic volatility

Phasic volatility was modeled with a Gaussian linear model with one predictor group (ASD = 1, COMP = -1).

Table S5: Estimates for the Phasic volatility model

	Estimate	Est.Error	Q2.5	Q97.5
Intercept	0.154	0.072	0.009	0.299
diagnosis1	0.101	0.070	-0.034	0.238

S4.2 Environmental tonic volatility

Modelled with a Gaussian linear model with one predictor group (ASD = 1, COMP = -1).

Table S6: Estimates for the Environmental tonic volatility model

	Estimate	Est.Error	Q2.5	Q97.5
Intercept	-2.247	0.222	-2.682	-1.811
diagnosis1	-0.034	0.165	-0.357	0.292

S4.3 Bernoulli model with HGF parameters

We also used an explorative Bernoulli model containint all scaled HGF parameters to predict group membership (ASD, COMP).

Table S7: Estimates for the explorative model including all HGF parameters

	Estimate	Est.Error	Q2.5	Q97.5
Intercept	0.000	0.272	-0.521	0.531
sbe1	-0.460	0.420	-1.297	0.355
sbe2	0.282	0.399	-0.483	1.085
sbe3	0.278	0.457	-0.593	1.195
sbe4	0.806	0.432	-0.021	1.679
sze	0.240	0.402	-0.551	1.035
som2	0.302	0.351	-0.380	1.000
som3	-0.139	0.352	-0.842	0.542

S4.4 Learning rate updates

Modelled with a lognormal linear mixed model with the predictors group (ASD, COMP), level (second- or third-level alpha) and change (prevolatile to volatile or volatile to postvolatile phase) and the random intercept for subject and slopes for level and change.

Table S8: Estimates for the Learning rate updates model

	Estimate	Est.Error	Q2.5	Q97.5
Intercept	-3.575	0.083	-3.742	-3.409
diagnosis1	0.168	0.085	0.001	0.333
level1	1.358	0.119	1.126	1.596
change1	1.109	0.045	1.017	1.198
diagnosis1:level1	0.029	0.121	-0.207	0.273
diagnosis1:change1	-0.090	0.045	-0.178	-0.001
level1:change1	-0.240	0.041	-0.320	-0.159
diagnosis1:level1:change1	0.118	0.041	0.037	0.198

Figure S7: Posterior predictive checks

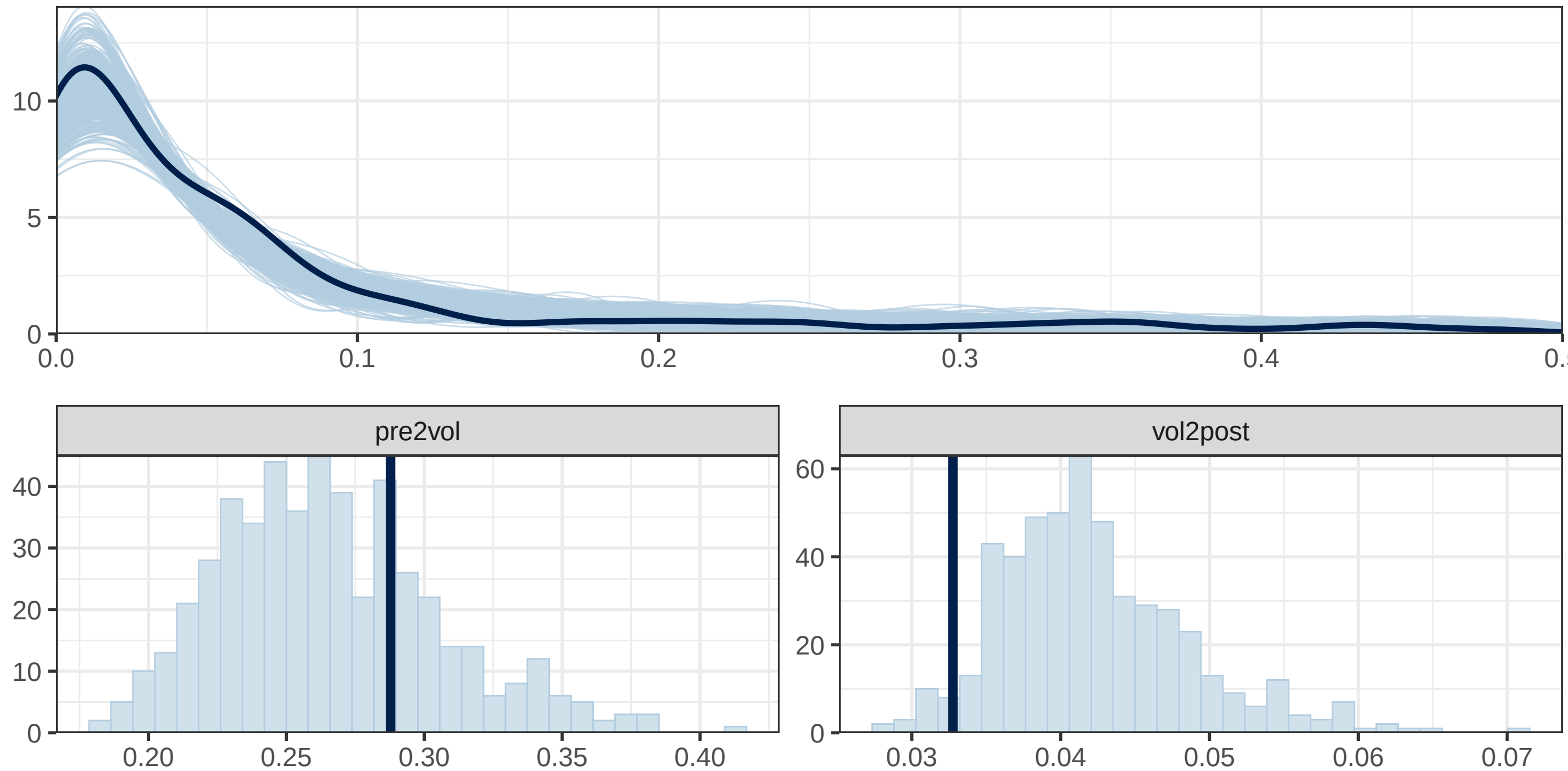

Table S9: ANOVA of ranked learning rate updates

	bf
level + change	105.308
level + change + level:change	105.274
diagnosis + level + change + level:change	104.709
diagnosis + level + change	104.631
diagnosis + level + change + diagnosis:change	103.743
diagnosis + level + change + diagnosis:change + level:change	103.633
diagnosis + level + diagnosis:level + change + level:change	103.524
diagnosis + level + diagnosis:level + change	103.471
diagnosis + level + diagnosis:level + change + diagnosis:change + level:change	102.352
diagnosis + level + diagnosis:level + change + diagnosis:change	102.351
diagnosis + level + diagnosis:level + change + diagnosis:change + level:change + diagnosis:level:change	101.547
level	51.219
diagnosis + level	49.990
diagnosis + level + diagnosis:level	48.643
change	23.304
diagnosis + change	21.886
diagnosis + change + diagnosis:change	20.537
diagnosis	-1.494

S5 Reaction times, pupil sizes and accuracies

S5.1 Reaction times

Reaction times were modelled with a shifted lognormal distribution and the predictors group (ASD, COMP), expected (yes, no), phase (prevolatile, volatile, postvolatile), difficulty (easy, medium, hard) and their interactions. All predictors were sum-coded. Furthermore, we added random intercept for subjects (slopes: expected, phase, difficulty and their interactions) and trials (slope: diagnosis). The full model can be downloaded from OSF: https://osf.io/wkgec.

Table S10: Fixed effects of the reaction time model

	Estimate	Est.Error	Q2.5	Q97.5
Intercept	6.174	0.029	6.117	6.230
diagnosis1	0.030	0.021	-0.010	0.070
expected1	-0.036	0.006	-0.048	-0.024
phase1	0.027	0.012	0.004	0.050
phase2	-0.007	0.008	-0.022	0.008
difficulty1	-0.028	0.008	-0.044	-0.013
difficulty2	-0.009	0.008	-0.024	0.007
diagnosis1:expected1	-0.001	0.004	-0.009	0.006
diagnosis1:phase1	0.000	0.010	-0.019	0.019
diagnosis1:phase2	-0.001	0.005	-0.011	0.010
expected1:phase1	-0.037	0.009	-0.054	-0.020
expected1:phase2	0.001	0.007	-0.013	0.015
diagnosis1:difficulty1	0.001	0.005	-0.008	0.010
diagnosis1:difficulty2	-0.004	0.005	-0.013	0.005
expected1:difficulty1	-0.015	0.008	-0.030	0.001
expected1:difficulty2	0.002	0.008	-0.014	0.018
phase1:difficulty1	-0.002	0.011	-0.024	0.020
phase2:difficulty1	0.008	0.010	-0.012	0.027
phase1:difficulty2	0.017	0.011	-0.005	0.040
phase2:difficulty2	-0.011	0.010	-0.030	0.008
diagnosis1:expected1:phase1	0.005	0.005	-0.005	0.015
diagnosis1:expected1:phase2	0.000	0.004	-0.008	0.008
diagnosis1:expected1:difficulty1	-0.001	0.005	-0.010	0.008
diagnosis1:expected1:difficulty2	0.003	0.005	-0.007	0.012
diagnosis1:phase1:difficulty1	0.006	0.007	-0.007	0.019
diagnosis1:phase2:difficulty1	-0.002	0.006	-0.014	0.010
diagnosis1:phase1:difficulty2	-0.007	0.007	-0.020	0.006
diagnosis1:phase2:difficulty2	0.006	0.006	-0.005	0.017
expected1:phase1:difficulty1	-0.008	0.011	-0.029	0.014
expected1:phase2:difficulty1	0.000	0.010	-0.019	0.019
expected1:phase1:difficulty2	0.008	0.011	-0.015	0.030
expected1:phase2:difficulty2	-0.004	0.010	-0.023	0.016
diagnosis1:expected1:phase1:difficulty1	0.003	0.007	-0.010	0.017
diagnosis1:expected1:phase2:difficulty1	0.001	0.006	-0.010	0.012
diagnosis1:expected1:phase1:difficulty2	0.000	0.007	-0.014	0.013
diagnosis1:expected1:phase2:difficulty2	-0.005	0.006	-0.016	0.006

Figure S8: Reaction times per subject

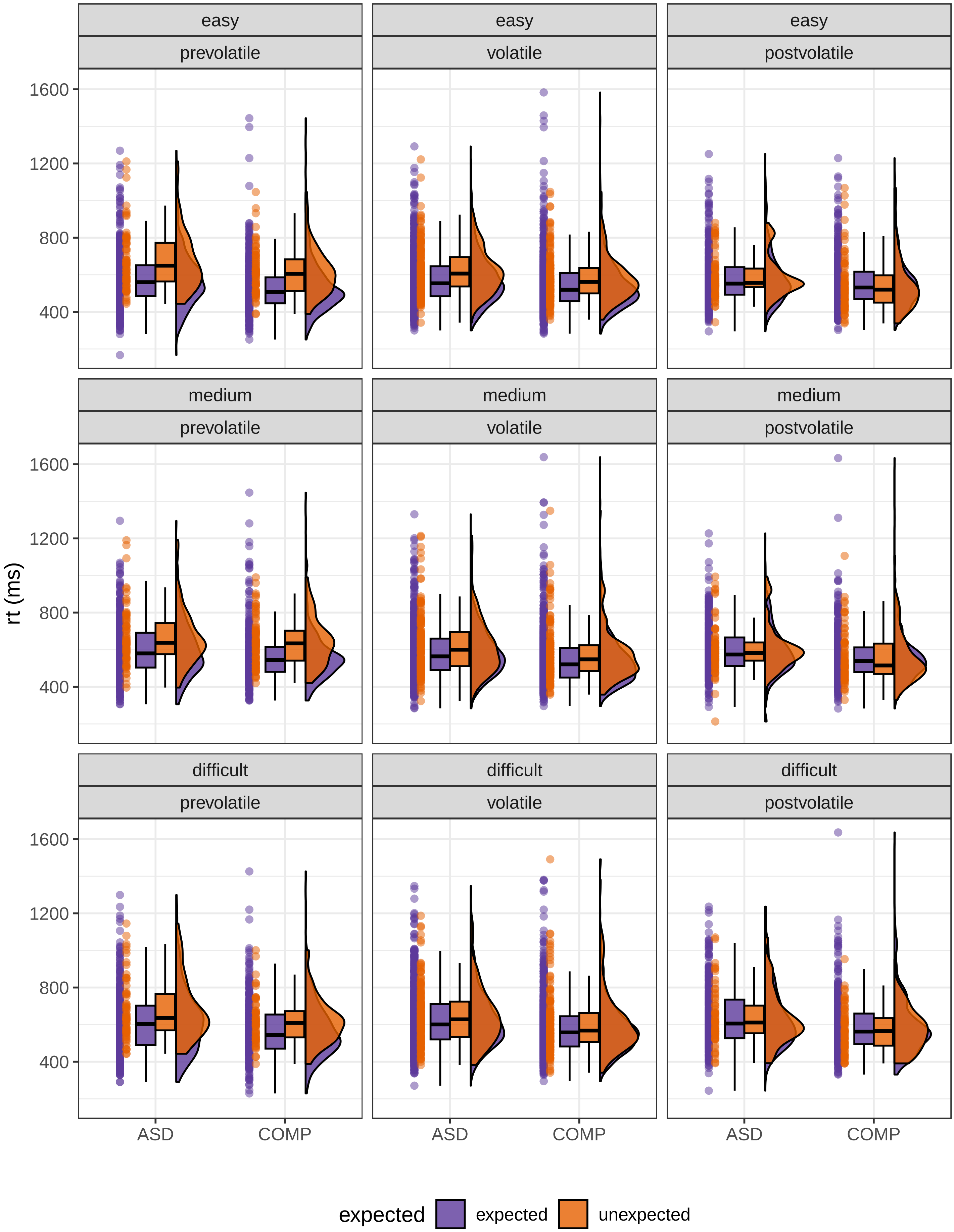

S5.2 Pupil sizes

Modeled with a Gaussian linear mixed model, including the predictors group (ASD, COMP) and expected (yes, no) with their interaction as well as the covariate rts and the random intercept for subjects.

Table S11: Fixed effects of the pupil size model

	Estimate	Est.Error	Q2.5	Q97.5
Intercept	0.085	0.013	0.059	0.109
diagnosis1	0.014	0.013	-0.012	0.040
expected1	-0.001	0.002	-0.005	0.004
rts	0.000	0.000	0.000	0.000
diagnosis1:expected1	-0.002	0.002	-0.006	0.003

Figure S9: Pupil size changes

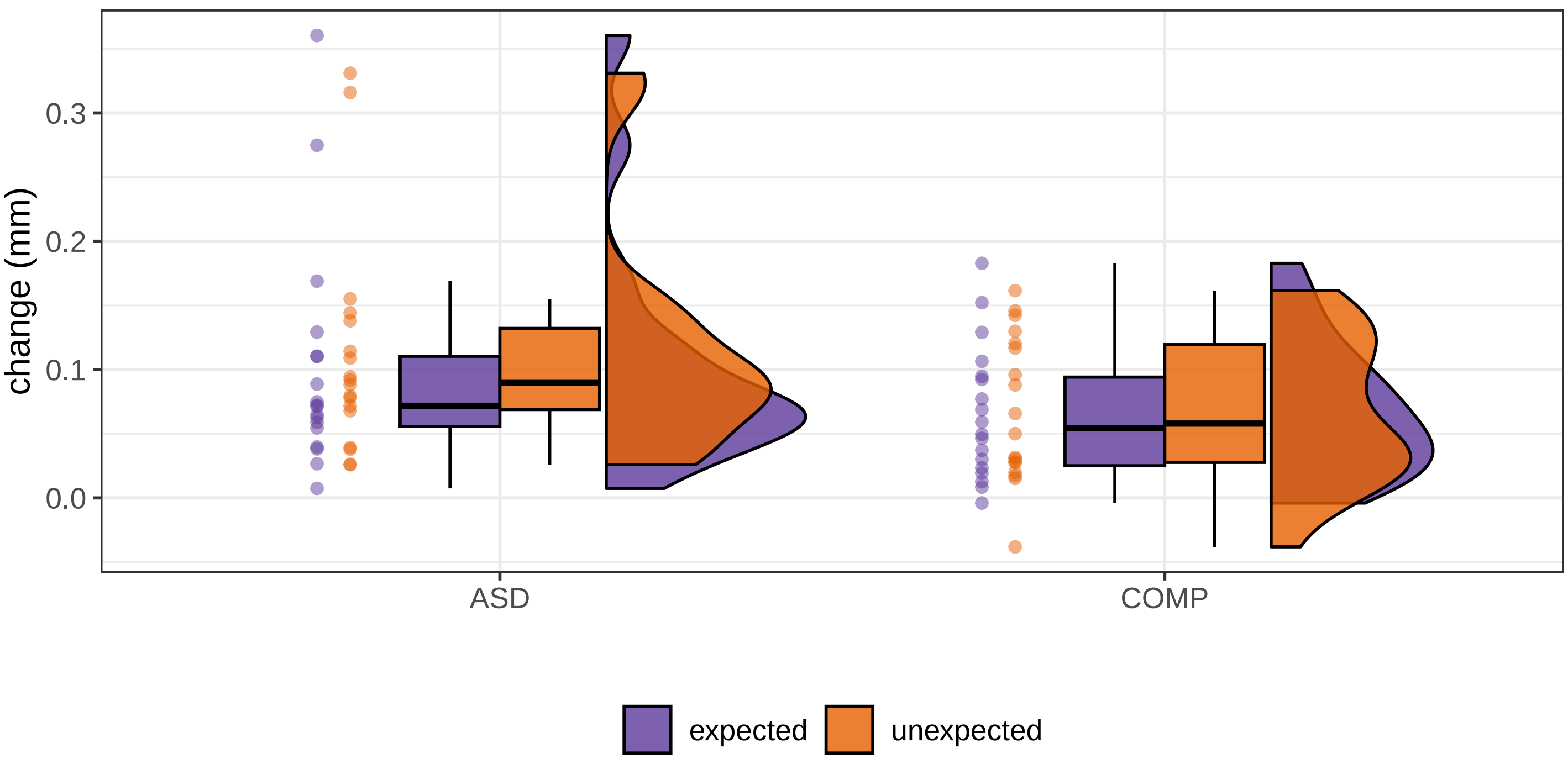

S5.3 Accuracies

ANOVA of the ranked accuracies, including the predictors group (ASD, COMP), expected (yes, no), phase (prevolatile, volatile, postvolatile), difficulty (easy, medium, hard) and their interactions. Since this results in a large number of models, we only print the best 5 models. The full ANOVA is shared on GitHub: https://github.com/IreneSophia/EMBA_PAL/blob/main/EMBA_PAL-ASD/_brms_models/aov_acc.rds.

Table S12: ANOVA of ranked accuracies

	bf
diagnosis + expected + difficulty	41.15
diagnosis + difficulty	40.83
diagnosis + expected + diagnosis:expected + difficulty	40.29
diagnosis + expected + difficulty + diagnosis:difficulty	39.74
diagnosis + difficulty + diagnosis:difficulty	39.39
diagnosis + expected + diagnosis:expected + difficulty + diagnosis:difficulty	38.93

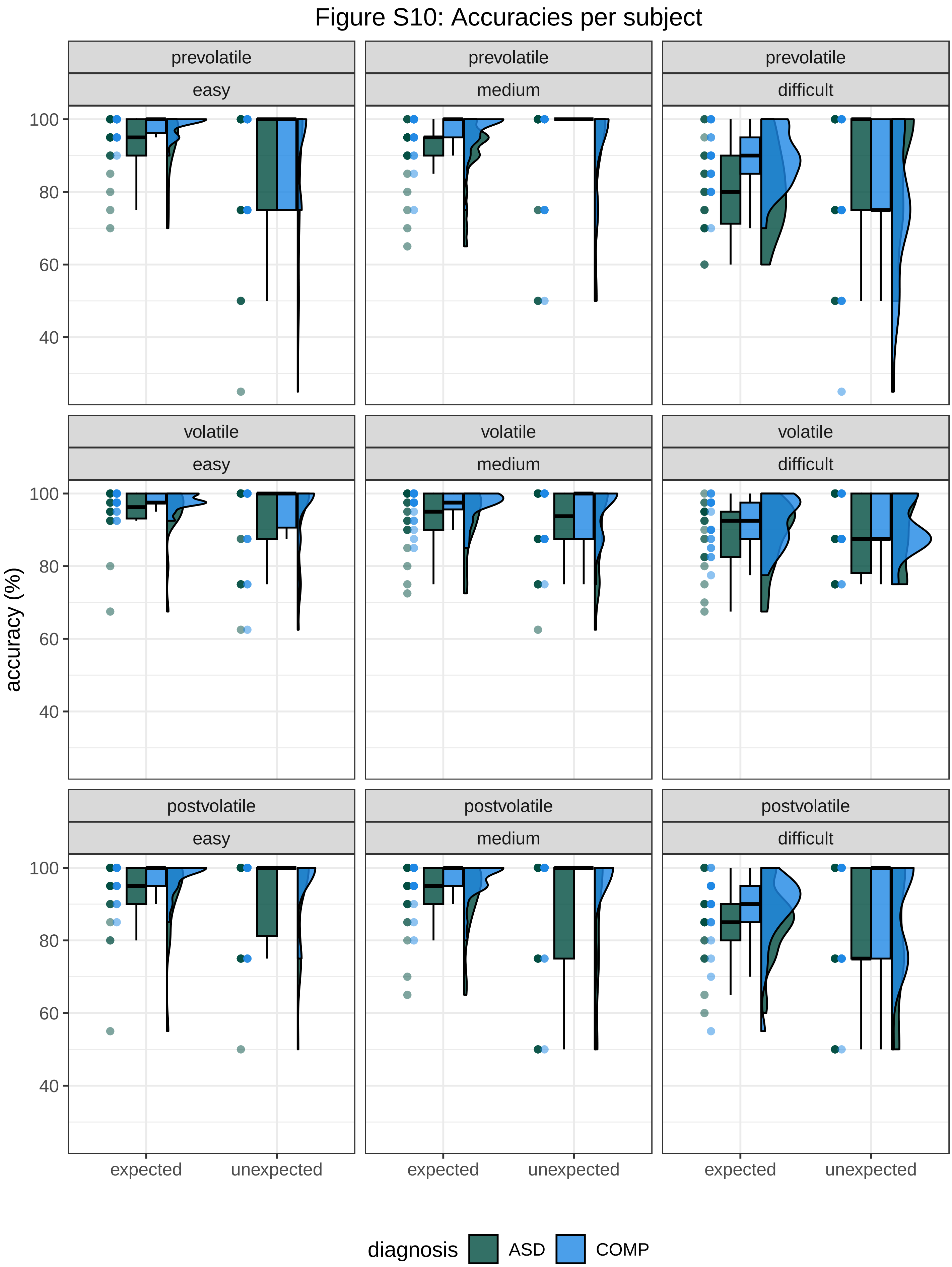
Figure S10: Accuracies per subject
prevolatile
easy
prevolatile
medium
prevolatile
difficult
volatile
easy
volatile
medium
volatile
difficult
postvolatile
easy
postvolatile
medium
postvolatile
difficult
accuracy (%)
100
80
60
40
expected
unexpected
diagnosis
ASD
COMP

S6 Learning rates proper

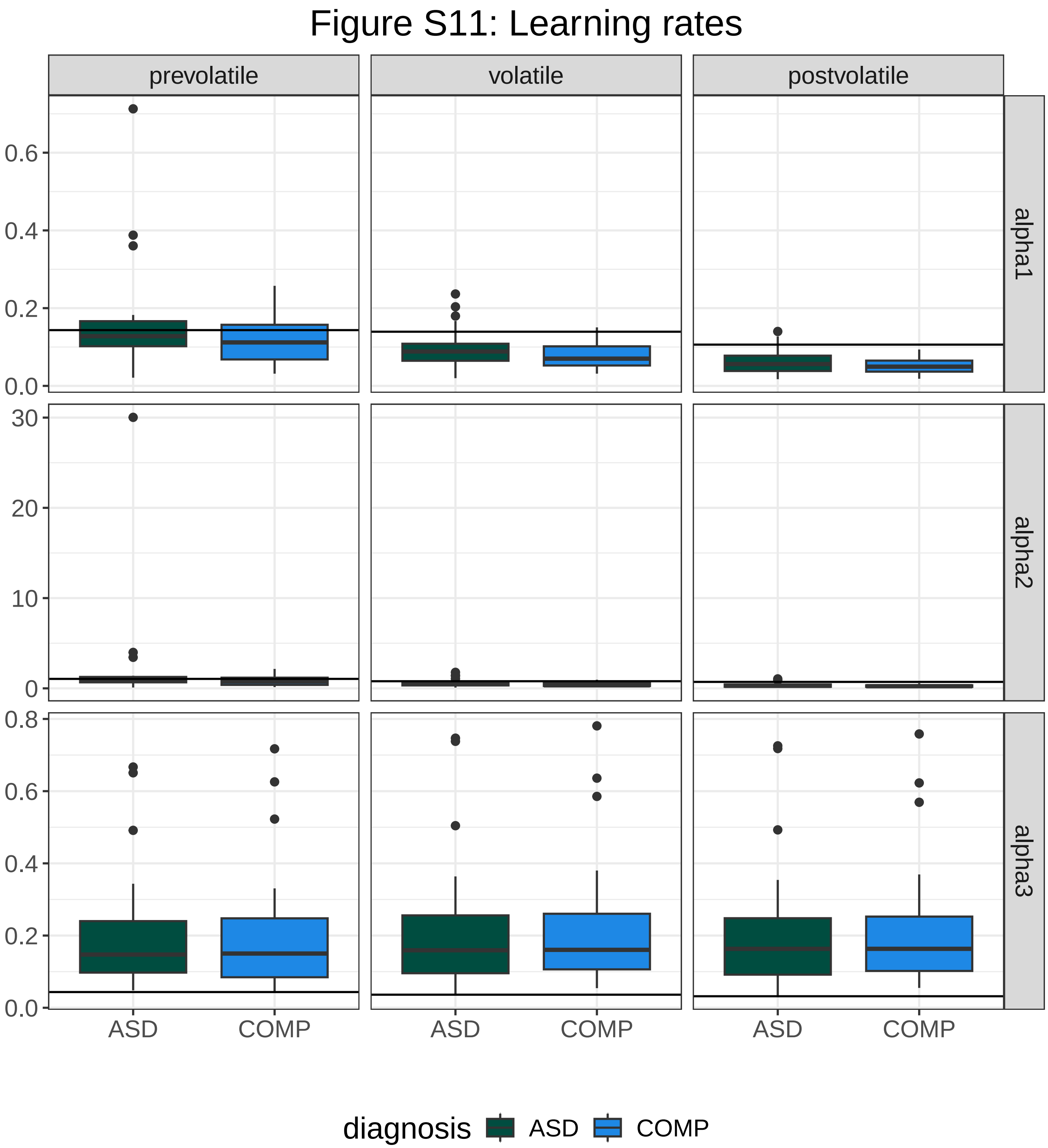

S7 Handedness and button assignment

We counterbalanced whether the left or the right button was to be clicked to indicate a positive valence. Here, we check whether there were any differences between groups in terms of handedness and button assignment. Information on handedness of one participant is missing, because they skipped this question.

```
##                          bf error                     time         code
## Non-indep. (a=1) -2.444793     0 Sat Apr 25 14:07:10 2026 62fa3c5cd315
```

Table S13: Handedness distribution across groups

diagnosis	handedness	n
ASD	beidh ndig	1
ASD	links	3
ASD	rechts	17
ASD	NA	1
COMP	links	3
COMP	rechts	19

Table S14: Handedness distribution across groups and key assignment

diagnosis	key_pos	handedness	n
ASD	left	beidh ndig	1
ASD	left	links	1
ASD	left	rechts	9
ASD	right	links	2
ASD	right	rechts	8
ASD	right	NA	1
COMP	left	links	2
COMP	left	rechts	9
COMP	right	links	1
COMP	right	rechts	10

S8 Learning rates based on Bayesian Observer

Table S15: Learning rate updates based on Bayesian Observer

level	change	value
2nd level learning rate updates	prevolatile to volatile	0.217
2nd level learning rate updates	volatile to postvolatile	0.059
3rd level learning rate updates	prevolatile to volatile	0.004
3rd level learning rate updates	volatile to postvolatile	0.001

S9 Exploration of gender differences

S9.1 Reaction times: expectancy effect

Figure S12: Expectancy effect in RTs including Gender

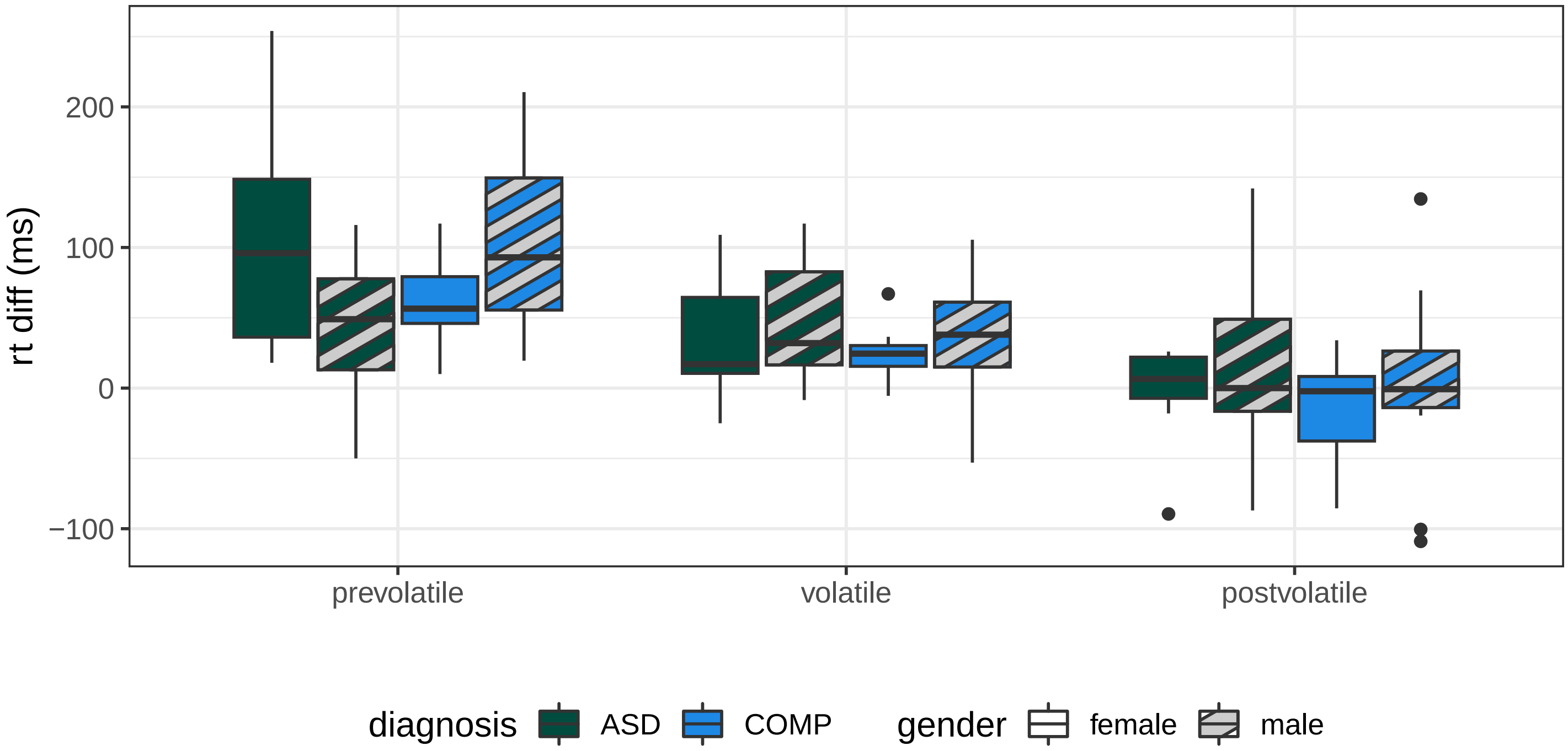

S9.2 HGF parameters

Table S16: Fixed effects of the Phasic environmental volatility model including Gender

	Estimate	Est.Error	Q2.5	Q97.5
Intercept	0.152	0.073	0.009	0.296
diagnosis1	0.104	0.070	-0.035	0.242
gender1	-0.043	0.070	-0.180	0.096
diagnosis1:gender1	0.014	0.071	-0.123	0.153

Table S17: Fixed effects of the Tonic environmental volatility model including Gender

	Estimate	Est.Error	Q2.5	Q97.5
Intercept	-2.256	0.221	-2.697	-1.823
diagnosis1	-0.029	0.165	-0.354	0.295
gender1	-0.068	0.164	-0.390	0.256
diagnosis1:gender1	0.100	0.166	-0.228	0.423

Figure S13: HGF parameter

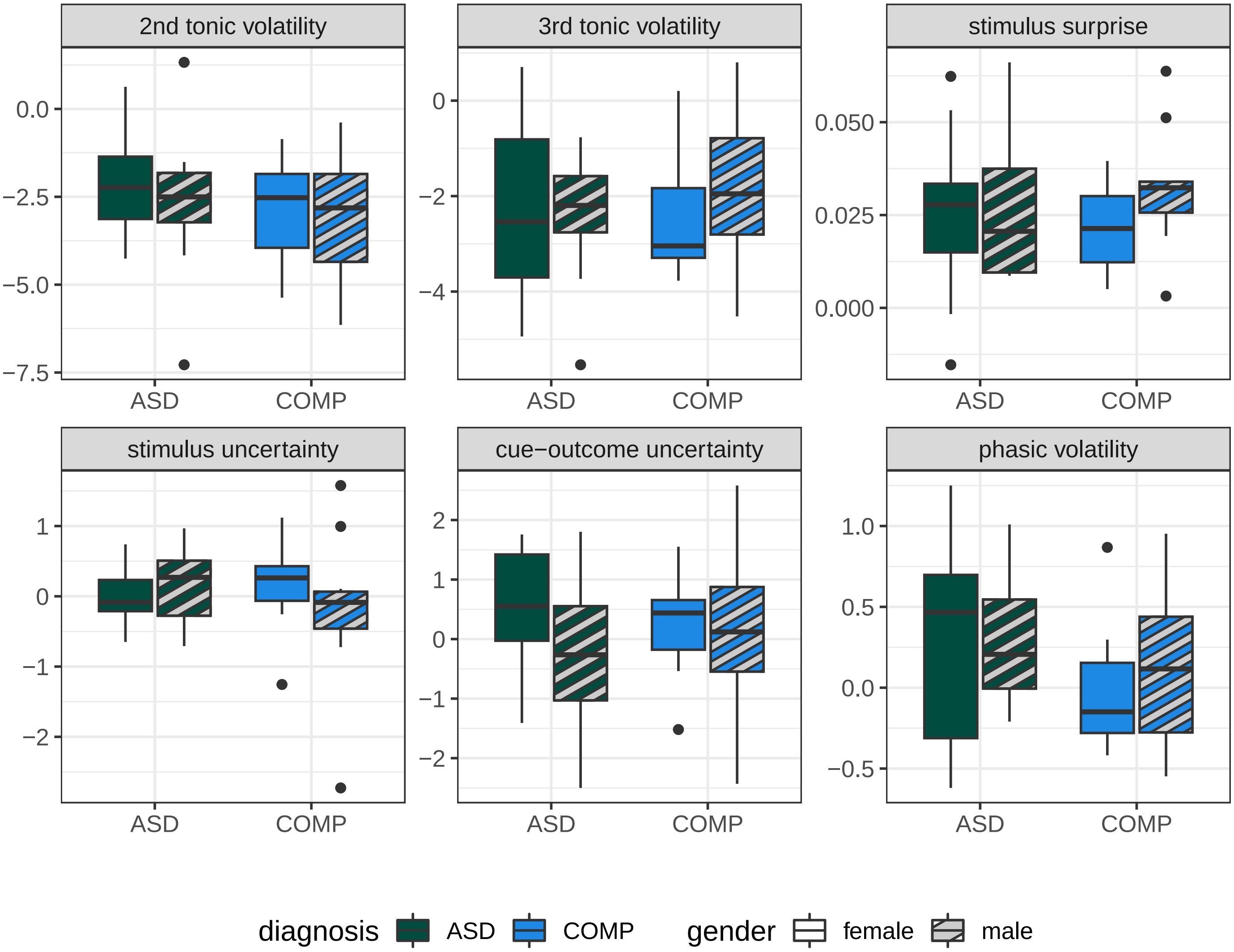

S9.3 Learning rate updates

Table S18: ANOVA investigating learning rate updates including the predictor Gender (5 best models)

	bf	bf.diff	bf.int
level + change	105.331	0.010	anecdotal evidence in favour of
level + change + level:change	105.321	0.630	anecdotal evidence in favour of
diagnosis + level + change + level:change	104.691	0.011	anecdotal evidence in favour of
diagnosis + level + change	104.679	0.862	anecdotal evidence in favour of
gender + level + change + level:change	103.818	0.040	anecdotal evidence in favour of
gender + level + change	103.777	0.124	anecdotal evidence in favour of

Figure S14: Learning rate updates

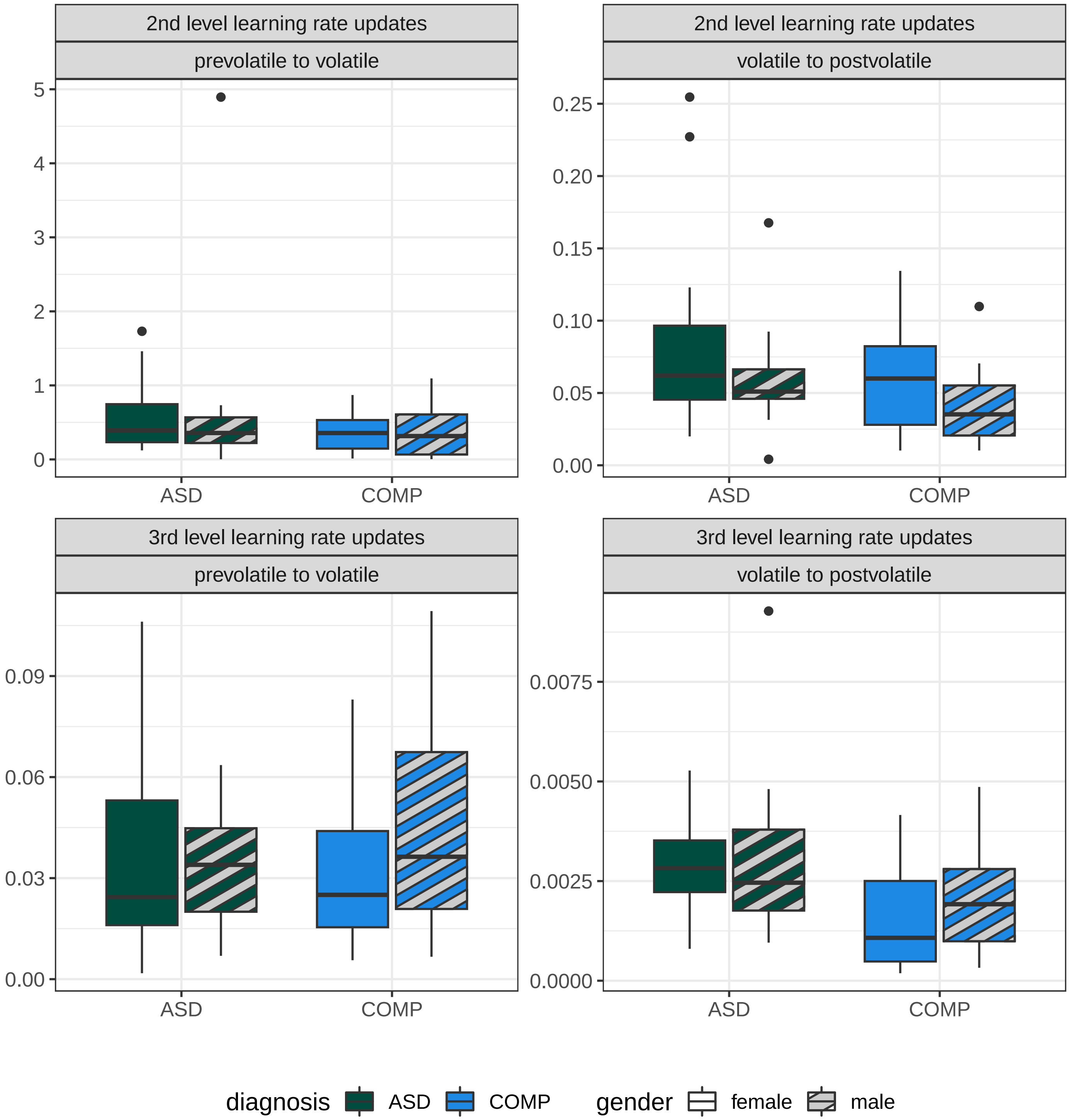

Supplement: Supplementary file 1 [file 13229_2026_730_MOESM1_ESM.pdf]
